# A TATA-box-binding protein binds single-stranded DNA in two modes: To poly(G) tracts and to flexible DNA regions

**DOI:** 10.1016/j.jbc.2025.108552

**Published:** 2025-04-27

**Authors:** Kieran Freitag, Melanie Marlow, Joella Joseph, Robert Ta, Jessica Krekhno, Evan Schuett, Ally Yang, Debashish Ray, Timothy Hughes, Steven Rafferty, Janet Yee

**Affiliations:** 1 Biochemistry and Molecular Biology Program, Trent University, Peterborough, Ontario, Canada; 2 Department of Molecular Genetics, University of Toronto, Toronto, Ontario, Canada

**Keywords:** transcription factor, *Giardia intestinalis*, DNA structure, base stacking energy, EMSA, protein binding microarray (PBM), computer modelling, G-quadruplex, AlphaFold3, parasite, protist

## Abstract

The TATA-box-binding protein (TBP) homolog from *Giardia intestinalis* (gTBP) is highly divergent, lacking key phenylalanine residues crucial for binding and unwinding double-stranded DNA. Surprisingly, we determined that gTBP exhibits unconventional DNA-binding properties and preferentially binds to single-stranded DNA (ssDNA) using a DNA-binding pocket that is narrower relative to other eukaryotic TBPs. Additionally, we showed that gTBP binds in two distinct modes, which we call the A and B modes, that are dependent on ssDNA sequence and protein concentration. For the A mode, gTBP binds as an oligomer to ssDNA that contains four or more consecutive guanine bases. For the B mode, using base stacking energy potentials between adjacent dinucleotides as a simple proxy for per-nucleotide flexibility, gTBP binds as a monomer to ssDNA in a manner that is dependent on DNA structural properties. To validate the latter concept, we designed *de novo* DNA sequences with base stacking energy profiles comparable to two DNA sequences that bind gTBP and showed that these designed sequences can compete for gTBP binding against the two original sequences. Overall, we present a potential new perspective on eukaryotic transcription regulation based on our findings around unconventional gTBP-ssDNA binding. A comprehensive understanding of the binding modes of gTBP could yield insights into *Giardia*’s biology and eukaryotic transcription in general.

*Giardia intestinalis* is a protozoan parasite that challenges our understanding of transcription in eukaryotes. Of the proteins involved in eukaryotic transcription, the TATA-box-binding protein (TBP) helps regulate the genes transcribed by three core RNA polymerases: I, II, and III (1). Transcription initiation is thought to occur when TBP recognizes and binds an AT-rich sequence on dsDNA called the TATA-box, inducing an 80-degree kink in the DNA (2, 3). However, this original model is incomplete as only ∼15% of gene promoters in eukaryotes such as humans and yeast (4), and none of those in *Giardia* (5) contain typical TATA-boxes. Resolving many of the issues of this early model, Chen and coworkers demonstrated that transcription initiation with RNA polymerase II can follow three separate paths determined by the promoter elements associated with the particular gene (6). Notably, one of these paths does not require a TATA-box promoter element. In all three cases, however, TBP is still required to induce a kink in the DNA, regardless of whether a TATA-box is present or not.

The regulation of transcription initiation in *Giardia* remains elusive. Although *Giardia* expresses a homolog of TBP (gTBP), it is highly divergent from other eukaryotes and contains substitutions in three of the four phenylalanine residues known to be crucial for binding DNA (5). *Giardia* also lacks homologs for TFIIA, TFIIB, and many TBP-associated factors that are seemingly required for transcription initiation (5). *Giardia* has a reduced genome (12.08 Mb) compared to many other eukaryotes and is missing several organelles (7). For instance, *Giardia* lacks peroxisomes, a classical Golgi apparatus, and has highly reduced mitochondria called mitosomes. These features are likely to have arisen from reductive evolution, and *Giardia* may retain only the most essential components in many of its cellular pathways (7, 8). Therefore, examining key processes such as transcription in *Giardia* has the potential to provide information about the fundamental nature of transcription for other eukaryotes as well.

In this study, we aim to further understand the role TBP plays in transcription in *Giardia* by examining its binding specificity toward DNA *in vitro*. We sought to accomplish this using electrophoretic mobility shift assays (EMSAs), high-throughput binding assays, and *in silico* ssDNA sequence analysis. Our results from a high-throughput DNA binding assay—protein binding microarray (PBM)—revealed a DNA consensus sequence that contains four central consecutive G nucleotides. We identified this consensus sequence in the previously characterized promoters of the histone H4 and histone H2B genes (9) and used the coding strand of these sequences as probes in EMSAs with recombinant gTBP. We verified that the central G nucleotides in these sequences are important for gTBP binding, but only when a high concentration of the protein was used; we refer to this binding of gTBP to DNA as the A mode. Interestingly, gTBP also binds to DNA that differs from the G-rich PBM motif when a lower protein concentration was used. Despite extensive analysis of the primary sequence of ssDNA representing other *Giardia* promoters, including those that have an AT-initiator, TATA-box-like sequences, or other AT-rich regions, we could not identify a consensus DNA sequence for the binding of gTBP in this mode, which we refer to as the B mode. However, when using the base stacking energy between adjacent dinucleotides, which has a particularly strong impact on the local flexibility of ssDNA (10, 11) we determined that structural features of ssDNA impact gTBP binding.

## Results

### *Giardia* encodes an unconventional TATA-box-binding protein

**Figure 1:**
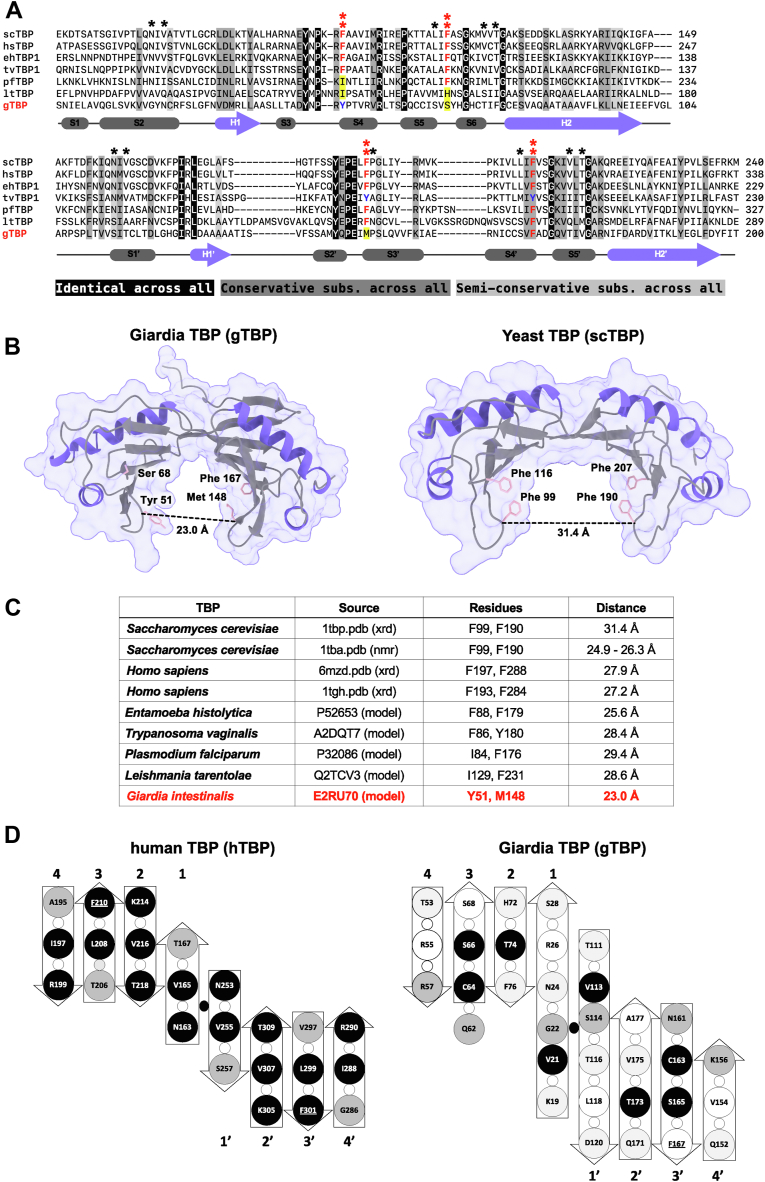
***Giardia* TBPsequence, but not structure,is divergent from TBP in other eukaryotes.***A*, sequence alignment of TBP orthologs from *Giardia lamblia* (gTBP) and other eukaryotes: *Saccharomyces cerevisiae* (scTBP), *Homo sapiens* (hsTBP), *Entamoeba histolytica* (ehTBP1), *Trichomonas vaginalis* (tvTBP1), *Plasmodium falciparum* (pfTBP), and *Leishmania tarentolae* (ltTBP). The secondary structure was determined from the gTBP amino acid sequence (H, α helix; S, β strand). Shaded columns represent different levels of conserved residues across the species: identical (*black*), conservative (*gray*), and semi-conservative (*light gray*). Phenylalanine residues identified as crucial for DNA binding, or their substitutions, are identified with *double asterisks* (∗∗) in *red*. At these ∗∗ positions, the phenylalanines are indicated in *red* font, conservative substitutions are in *blue* font, and nonconservative substitution are highlighted. Other residues identified for double stranded TATA-box recognition, or their substitutions, are identified with single asterisks (in *black*). *B*, tertiary structure for *Giardia* TBP (gTBP) and *Saccharomyces cerevisiae* (scTBP). The predicted model for gTBP (GL50803_001721) was determined using AlphaFold 2.0. The structure has an average per-residue confidence score > 90, indicating a high estimated modeling accuracy (Fig. S1). The structure for scTBP was determined by X-ray crystallography (PDB: 1YTB). *C*, the distances between the alpha carbons of the conserved pair of phenylalanine residues at the stirrups of the TBP structures, or their equivalent substituted amino acids, are used as the width of the underside of the saddle that is the DNA binding domain. A line connecting these residues in the gTBP and scTBP structures is shown in part *B* above as an example. The information in the source column of the Table indicates whether the experimental structures were obtained by X-ray diffraction (xrd), nuclear magnetic resonance spectroscopy (nmr) or predicted by AlphaFold 2.0 (model). *D*, a comparison of the beta sheets of human TBP (*left*) and *Giardia* TBP (*right*) viewed at the underside of the saddle structure. Individual beta strands are numbered 1 to 4 and their symmetry-related counterparts are numbered 1′-4’. The oval dot in the center of each TBP structure represents the pseudo-C2 rotational axis that is directed towards the viewer. Residues at symmetry related positions in the two halves of the saddle are indicated by *circles*: *black shading* denotes residues that are identical at symmetry-related positions in the two halves of the saddle; *grey* shading denotes residues that are similar, and *white* denotes residues at symmetry-related positions that are not similar. Conserved phenylalanine residues at the TBP stirrups are underlined. *Small white circles* indicate residues whose side chains are directed away from the face of the saddle that contacts nucleic acids.

*G. intestinalis* has a single gene encoding TBP (gTBP; GL50803_001721), and its predicted amino acid sequence was aligned with TBPs from *Saccharomyces cerevisiae* (scTBP; 20.2%), *Homo sapiens* (hsTBP; 20.7%), *Entamoeba histolytica* (ehTBP1; 21.5%), *Trichomonas vaginalis* (tvTBP1; 22.2%), *Plasmodium falciparum* (pfTBP; 19.3%), and *Leishmania tarentolae* (ltTBP; 21.9%) in Figure 1*A*. The yeast and human TBP sequences were chosen as representatives of canonical TBPs while the remaining sequences are those from other protozoan parasites that have highly divergent TBPs (12). Overall, gTBP shares 19% to 22% identity with the TBPs of the aforementioned species, with specific percentages shown in brackets. Four phenylalanine residues in TBP that are considered to be important for intercalating with dsDNA to induce a kink in the DNA helix (2, 3) are substituted in three positions in *Giardia*: two are non-conservative (F68S, F148M) and one is conservative (F51Y) (5). Additionally, *Giardia* contains substitutions in 10 of the 11 other residues identified by Juo *et al.* (13) for directly interacting with the bases of TATA-box dsDNA *via* van der Waal’s contacts: N22G, V24N, L66S, V74T, T76F, N114S, V116T, L165S, V173T, and T175V (P149 is unchanged). These 15 residues (2 Phe pairs, 11 others) are thought to play a large role in the recognition and specificity of dsDNA-TBP interaction at the TATA-box (13).

The predicted gTBP structure has dyad symmetry with two α-helices and four to five β-sheets in each half (Fig. 1*B*, left). Despite the high divergence and substitutions of key residues in the gTBP sequence, the predicted structure of gTBP has the saddle shape of other gTBPs, including the one from *S. cerevisiae* (Fig. 1*B*, right). In the *S. cerevisiae* TBP, the top or convex side of the saddle is where other TBP-associated factors (TAFs) will bind, while the bottom or concave side of the saddle is where the protein straddles the DNA, with extensions on each side to form the stirrups (2, 3, 14).

The AlphaFold2 model of the gTBP model structure has a per-residue confidence (pLDDT) of greater than 88% and is especially high in the residues of the saddle itself. For most of the model, the predicted aligned error (PAE), which is the measurement of the confidence of the predicted structure, is very high within each half of the saddle and high between these halves (Fig. S1). The gTBP model retains the beta sheet saddle structure common to other TBPs, but interestingly, the two halves of the gTBP saddle are drawn inward, resulting in a narrower channel. The central beta strands of the saddle of gTBP (residues 16–28, 13 residues and 111–120, 10 residues) are longer than those of human TBP (residues 160–169, 10 residues and 253–259, seven residues) and are predicted to make more extensive contacts with their adjacent antiparallel beta strand and overlaying alpha helix. This imparts a twist in the structure such that the remaining three strands of each half of the saddle are drawn inwards in gTBP. This seems unlikely to be an artefact of model-building, as the predicted structures of other protozoan TBPs retain the wider saddle shape (Fig. S2). To compare the saddle widths, we measured the distance between the alpha carbons corresponding to the conserved pair of phenylalanine residues in the stirrups of the saddle among the TBPs shown in the alignment (Figs. 1*C* and S2). The distance between these pair of residues in gTBP is 23.0 Å, compared to an average distance of 27.7 ± 2.0 Å for the other TBPs.

The two halves of the saddle of TBPs have dyad symmetry with respect to the arrangement of their beta strands showing that it is structurally a pseudo-dimer. In human TBP, this symmetry extends down to the level of the amino acid side chains on the concave surface that serves as the nucleic acid binding site; nine of the 12 residues in one half of the saddle have an identical symmetry-matched residue in the other half of the saddle, and the remaining positions have similar residues (Fig. 1*D*). For example, comparison of human TBP β-strand 4 to strand 4′ show that I197 is symmetry-matched to I288, and R199 to R290. The nucleic acid binding surface of the saddle of gTBP has much lower symmetry; only four of the 16 residues in one half the saddle have an identical symmetry-matched partner in the other half, and while eight residues in one half of the saddle have similar residues at analogous positions in the other half, the remaining positions have residues that are dissimilar, which is not seen with human or yeast TBP. For example, comparison of gTBP β-strand 4 to strand 4′ show R57 is similar to K156, but R55 is dissimilar to V154. We also note that gTBP beta strand 2' (residues 171–177) is longer than its symmetry-matched strand 2 (residues 72–76), which further lowers the symmetry of the saddle. A saddle structure with pseudo-C2 symmetry is consistent with binding a partner with dyad symmetry such as double-stranded DNA. This is seen with human and yeast TBPs in which the binding of two pairs of symmetry-matched and highly conserved phenylalanine residues to the minor groove of dsDNA introduce a kink into its structure. The lack of three of these phenylalanine residues in gTBP as well as the lower symmetry and narrowness of its saddle point to a different binding targets and function for this *Giardia* protein.

### Nuclear localization of *Giardia* TBP

**Figure 2:**
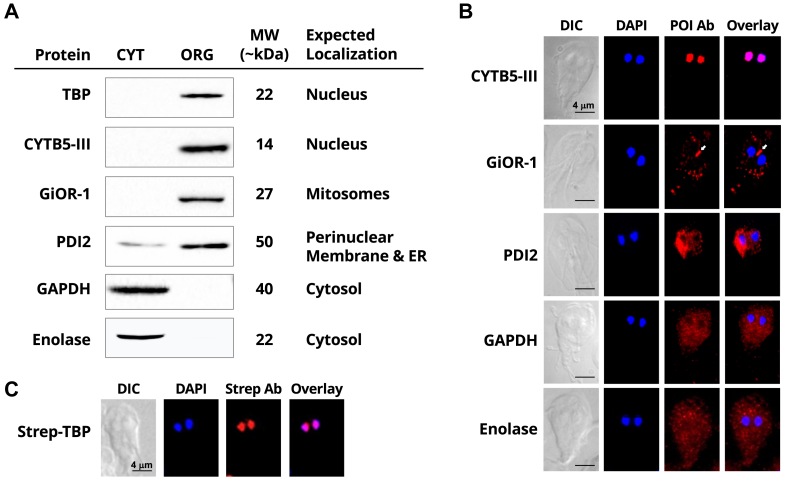
**Localization of *Giardia* proteins.***A*, a crude subcellular fractionation was performed on *Giardia* trophozoites to obtain a fraction enriched in cytosolic proteins (CYT) and a fraction enriched in organellar proteins (ORG). Proteins from both fractions were analyzed by Western blot with antibodies against TBP and five other proteins in *Giardia*. The predicted MW in kDa for each protein is shown and as well as the expected localization of each protein. *B*, immunofluorescence microscopy assays were performed to examine localization of *Giardia* proteins with the same antibodies used for subcellular fractionation. POI Ab represents the fluorescent image for the protein of interest detected by the corresponding antibody. The location of the central mitosomes between the two nuclei in the trophozoite are indicated by the white arrow in the results for the GiOR-1 antibody. *C*, immunofluorescence staining of a StrepII-tagged gTBP expressed in *Giardia* trophozoites with an anti-StrepII antibody.

TBP in *Giardia* is expected to be localized to the nucleus based on its proposed function in transcription and the nuclear localization of TBPs in other eukaryotes (15, 16). We examined gTBP localization in crude subcellular fractions of *Giardia* proteins. Here, immunoblot analysis of both cytosolic and organelle fractions of *Giardia* cells with an anti-gTBP antibody shows that gTBP is found only in the organelle fraction (Fig. 2*A*). The robustness of this fractionation procedure is supported by the correct localization of GiOR-1, the *Giardia* NADPH-dependent Tah18-like oxidoreductase, to mitosomes as previously described (17). Additionally, protein sulfide isomerase 2 (PDI2), an enzyme associated with ER and perinuclear membranes in immunofluorescence microscopy in *Giardia* (18, 19), is detected in organelle and cytosolic fractions. The localizations of these proteins are further validated using the same antibodies in immunofluorescent microscopy assays (Fig. 2*B*). Cytochrome *b*_5_ isotype III (20) displays exclusive nuclear localization with this method (Fig. 2*A*) as well as with immunofluorescence microscopy (Fig. 2*B*). However, we were not able to detect gTBP by immunofluorescence microscopy, likely due to deficiencies in the available antibody. To circumvent this issue, a StrepII-tagged gTBP was expressed in *Giardia* and detected using an anti-StrepII antibody. Here, strong localization of StrepII-gTBP was observed in both nuclei of *Giardia* trophozoites (Fig. 2*C*). Collectively, our results confirm that *Giardia* TBP localizes to the nucleus.

### *Giardia* TBP preferentially binds single-stranded DNA

**Figure 3:**
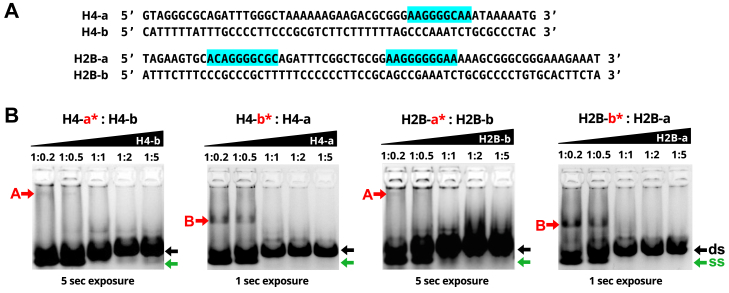
***Giardia*****TBP prefers to bind to single stranded DNA for two *Giardia* promoter sequences.***A*, sequences of coding (a) and non-coding (b) strands of DNA probes used. Highlighted regions represent matches to the motifs determined from the protein binding microarray analysis (Fig. 5). *B*, representative EMSA (n = 2) showing gTBP interacting with double stranded H4 or H2B probes made using differing ratios of complementary DNA strands. Only one of the two DNA strands is fluorescently labeled, as indicated by ∗ and in red. A constant 0.1 pmol/μl of the fluorescently labelled strand was annealed with an increasing amount of the corresponding complementary unlabeled sequence in each reaction, as indicated by the molar ratios above each lane. The *red* arrow indicates the shifted complex *A* and *B* formed on the a-strand and b-strand probes, respectively. At the *bottom* of each gel, the *lower green arrow* indicates the position of the unbound ssDNA probe, and the *upper black arrow* indicates the position of the unbound dsDNA probe on each gel.

We initially tested the ability of gTBP to bind DNA representing the promoter of the *Giardia* histone H4 (GL50803_00135002 and 00135003) and H2B (GL50803_00121045 and 00121046) genes in EMSAs due to the availability of these labeled probes from previous experiments (9). We observed binding of gTBP to single-stranded DNA probes corresponding to both strands of these promoters, with the non-coding (b-) strands binding better than the coding (a-) strands (Fig. 3). We discovered that gTBP binds poorly to dsDNA of these promoters when titrating increasing molar amounts of an unlabeled DNA strand that is complementary to the fluorescently labelled probe DNA in EMSAs (Fig. 3). Here, the shifted bands (see red arrows labelled A and B) representing the gTBP-DNA complexes decrease for all four labeled probes (H4-a∗, H4-b∗, H2B-a∗, H2B-b∗) when an increasing amount of the complementary and unlabeled DNA strand was added and annealed before the addition of the protein. The transition of ssDNA probe to dsDNA probe across the samples in each gel could be observed by the slight up-shift for corresponding bands (see green and black arrows labelled single-stranded [ss] and double-stranded [ds], respectively).

**Figure 4:**
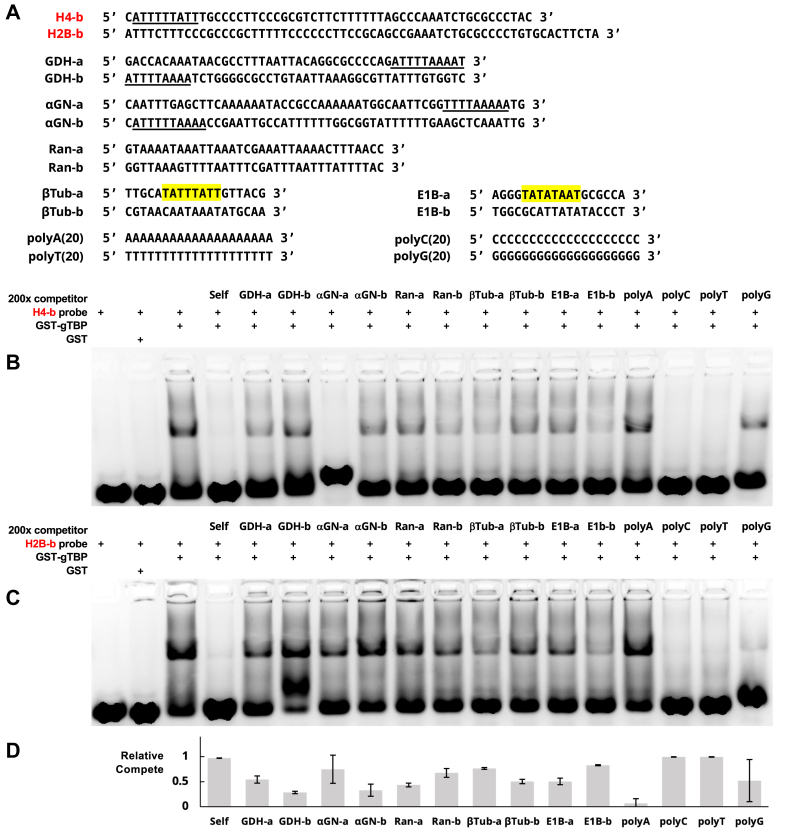
***Giardia*****TBP does not bind exclusively to canonical TATA boxes and other AT-rich sequences on single-stranded DNA.***A*, Sequences of DNA probes and competitors used. *B*, representative EMSA (n = 2) with single-stranded H4-b DNA probes against various single-stranded competitors (2.1 s exposure). Underlined regions represent the AT-rich initiator sequences, and highlighted regions represent sequences matching the canonical TATA box. The second lane contains the DNA probe and recombinant GST to demonstrate a lack of gTBP binding to GST alone. *C*, similar to (*B*), with single-stranded H2B-b as the DNA probe. *D*, quantification of binding to competitors on EMSAs for both DNA probes (n = 2 each probe; n = 4 in total). Relative strength of competition is determined with respect to the lane containing only the DNA probe and gTBP. Relative binding values range from 0 (no competition/low binding affinity) to 1 (complete competition/high relative binding affinity). Error bars represent standard deviation.

We then tested ssDNA corresponding to the promoter regions of four other *Giardia* genes, the adenovirus E1B promoter containing a canonical TATA-box, as well as poly-A, -C, -T, and -G sequences as competitors against the H4-b and H2B-b probes for binding to gTBP (Fig. 4). The promoter regions for the glutamate dehydrogenase GDH (GL50803_0021942) and α-Giardin (GL50803_0011654) genes contain the AT-rich initiator, and the Ran promoter (GL50803_0015869) contains several AT-rich sequences, which were previously shown to be important promoter elements in *Giardia* (21, 22). Despite these similarities, the ssDNA corresponding to these promoters competed to different extents (Fig. 4). The Adenovirus E1B promoter and the *Giardia* β-tubulin (GL50803_00136020) promoter both contain sequences matching the canonical TATA-box, but they show differential competition for binding to gTBP. Finally, poly(C) and poly (T) are good competitors, but poly(A) and poly(G) are not. The reduction of shifted complex with the poly(G) sample with the H2B-b probe (Fig. 4*C*, last lane) is likely due to the reduction of single-stranded H2B-b probe due to the complementary base pairing of several poly(C) or C-rich regions in the probe with the unlabeled poly(G) sequence rather than through direct competition with the poly(G) sequence. The a-strand of the α-Giardin promoter competes for gTBP binding only with the H4-b probe and not the H2B-b probe. Since a slightly shifted band above the band corresponding to the unbound probe is observed for this sample with the H4-b probe (Fig. 4*B*, see lane labeled αGN-a), it is possible that the α-Giardin sequence is interacting with the H4-b sequence so that there is less single-stranded DNA probe in this sample despite an apparent lack of complementary bases shared between these two sequences.

Since gTBP has a strong preference for ssDNA and the predicted structure of gTBP has a narrower DNA-binding domain than other TBPs, we assessed the ability of AlphaFold3 to model gTBP binding to ssDNA and dsDNA (Fig. S3). As a control, we modeled the binding of human TBP (hTBP) to the dsDNA oligos containing the E1B TATA sequence. Here, we obtained high confidence models for binding (ipTM 0.95, pTM 0.96) that are consistent with the structure of the hTBP-TATA complex reported by Dickerson and colleagues (1tgh.pdb) (13). In contrast, all attempts to model gTBP binding to dsDNA (E1B TATA, a CT-rich sequence, and other sequences) were unsuccessful (ipTM 0.07, pTM 0.79). The inability of AlphaFold3 to successfully model an interaction between gTBP and dsDNA is consistent with our earlier EMSA experiments and supports the idea that gTBP does not bind to dsDNA (Fig. 3). In contrast, models with good ipTM and pTM scores were obtained for gTBP binding to ssDNA. We found that the most significant parameter that affected model quality was the length of the ssDNA used for generating the complex, and not the sequence of the DNA. In general, ipTM and pTM scores of 0.8 or higher were usually obtained for model protein-ssDNA complexes of gTBP and hTBP with oligonucleotides containing up to 11 bases (Fig. S3 and File S5). Each input protein and nucleic acid query into AlphaFold gives five models in the default setting. In general, all five models generated for hTBP with either ssDNA or dsDNA are very similar, with only slight conformational changes in the protein and/or DNA (data not shown). However, the five models generated for gTBP with the CT-rich central region in the b-strand of the histone H4 promoter showed much greater conformational changes (Fig. S3*A*). For example, model “0” for gTBP with the CT-rich ssDNA, the two halves of the protein are drawn in closer to each other and the ssDNA is more compressed within the binding pocket, while in model “3”, the two halves of the gTBP are further apart and the ssDNA is more elongated in the binding pocket. These variations are also observed in models of gTBP with other ssDNA, including polyC, polyT, and the E1B TATA (data not shown). These results suggest flexibility in the conformation of gTBP when it is interacting with ssDNA compared to hTBP’s interaction with either ssDNA or dsDNA.

Since gTBP binds ssDNA, we questioned if it could also bind RNA, as some RNA-binding proteins have been shown to bind ssDNA (23). Thus, we used a high-throughput RNA-binding assay, RNAcompete, to determine if gTBP binds RNA. Here, gTBP was incubated with a diverse RNA library comprised of approximately 241,000 unique sequences (individual 7-mers are represented at least 155 times), and gTBP-bound RNA was eluted, labeled with fluorescent dye, and analyzed following microarray hybridization. No detectable interactions between gTBP and RNA were observed (Fig. S5).

**Figure 5:**
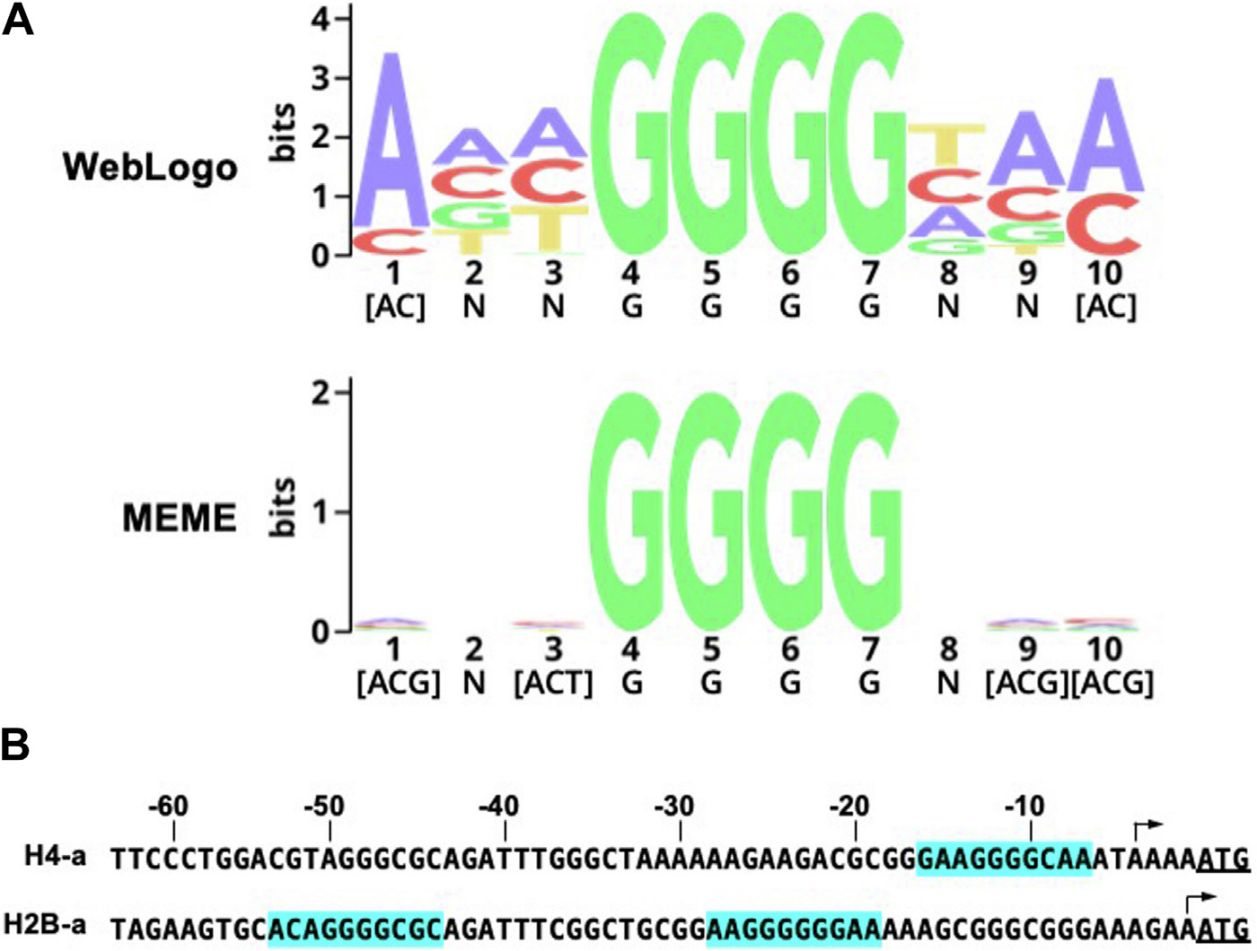
**DNA motifs were determined using a protein binding microarray(PBM)with*Giardia*TBP against single-stranded DNA.***A*, motifs were generated using the standard procedures for two web-based motif-generation tools: WebLogo and MEME. *B*, identification of PBM consensus sequence in promoter regions in the coding or a-strand of genes encoding histone H4 and H2B. The numbers at the top indicate the nucleotide positions in the DNA sequences relative to the translation start codon (underlined) for each gene. The bent arrows indicate the start of transcription for each gene determined previously.

Next, to systematically interrogate gTBP binding to ssDNA, we used gTBP in protein-binding microarrays (PBMs) to obtain DNA-binding data from diverse DNA probes and subsequently generate prospective ssDNA-binding motifs (24). The PBMs are composed of two distinct array patterns (ME and HK) containing approximately 41,000 oligonucleotides that are 35 nucleotides long, where every DNA 7-mer is represented at least 128 times (25, 26). Processed data from PBM experiments were analyzed using two different motif generating programs, WebLogo and MEME (Fig. 5*A*) and both generated motifs containing four continuous G nucleotides. We used the WebLogo and MEME motifs as well as GGGG alone to search for the gene promoter regions in the *Giardia* genome. Since *Giardia* promoter sequences are known to be short regions ranging between 50 and 60 bp and located immediately upstream of the start of the coding region (9, 21, 22, 27, 28), we searched all regions 75 bp upstream of the translation start codon of each gene in the *Giardia* genome, excluding 320 pseudogenes and three rRNA genes with corrupted entries (https://giardiadb.org). Out of the remaining total 5068 genes in the *G. intestinalis* WB isolate 2019 reference genome, we identified 173 genes with the WebLogo motif, 239 genes with the MEME motif, and 551 genes with GGGG contained within 75 bp upstream of the start of the coding region (File S4). The frequency of each motif was approximately equal on each DNA strand (File S4). The coding, or a-strands of the promoters of the histone H2B and H4 genes were among the *Giardia* genes with matches to the PBM logos. H2B-a has two independent matches to the WebLogo motif and H4-a has one match to the MEME motif (Fig. 5*B*). These regions were previously shown to contain the minimal promoter regions for these genes by luciferase reporter assays, and the transcription start sites have been mapped by 5′RACE and primer extension (9).

We previously observed gTBP binding to both the a- and b-strands of H4 and H2B in Figure 3. However, the gTBP complexes observed between these strands migrated to different positions on the gels and have different band intensities. For instance, to observe gTBP complexes with the a-strands, we required an increased exposure time of the gel compared to that of the b-strands. Since only the a-strands of these probes contain matches to the PBM sequence (Fig. 5*B* and File S4), which is presumably the sequence with the greater affinity to gTBP, we were intrigued that gTBP seems to bind to the b-strands more strongly and gives a lower migrating shifted complex compared to the a-strands. We explore this phenomenon through the experiments outlined in the next section.

### *Giardia* TBP binds single-stranded DNA in two distinct modes based on DNA sequence and protein concentration

**Figure 6:**
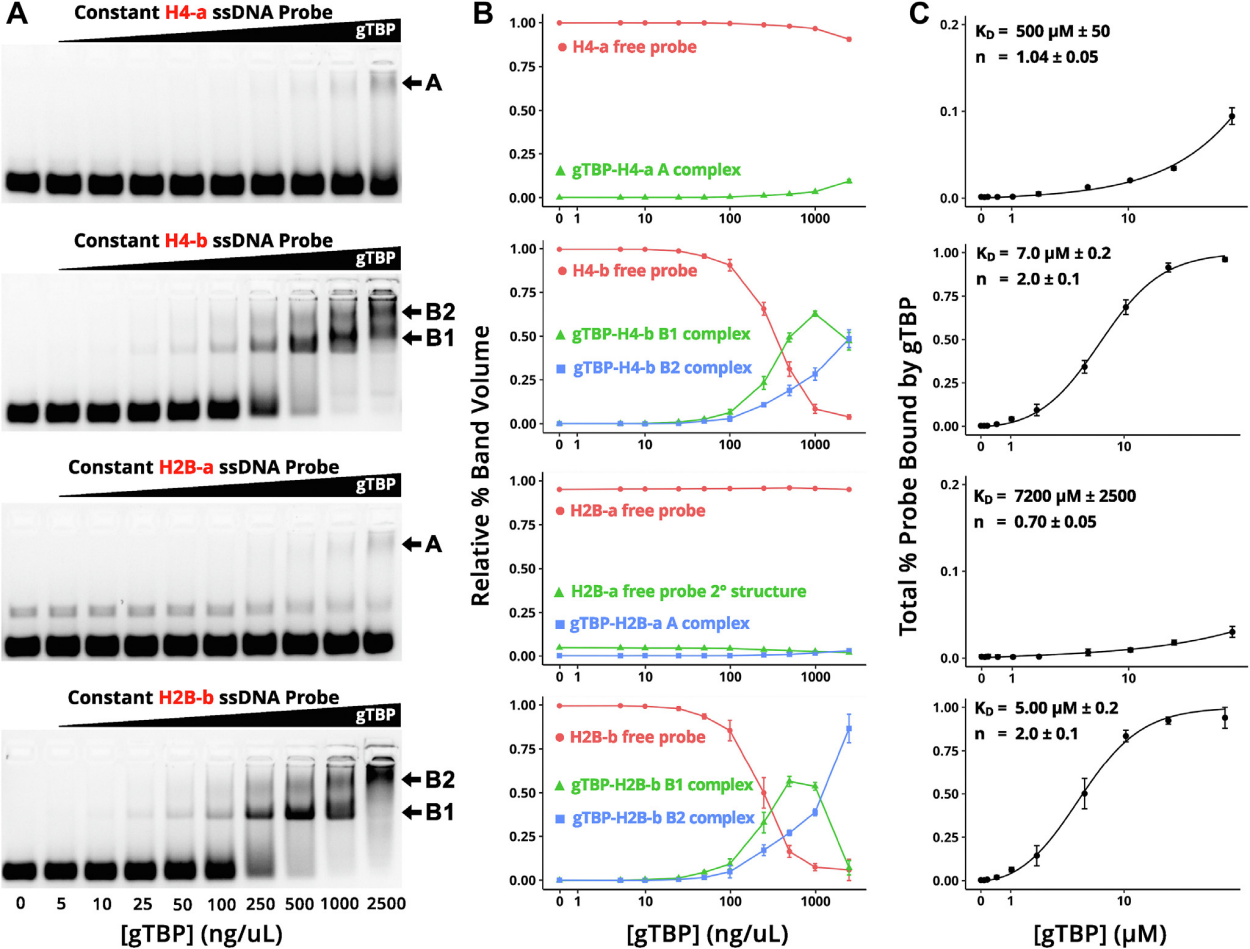
***Giardia*****TBPbinds withstrongeraffinity to the non-coding (b) strands of the H4 and H2B promotersand exhibits various modes of binding to the DNA probes.***A*, representative EMSA (n = 4) using a constant amount of the specified labelled DNA probe with an increasing amount of gTBP from 0 to 2500 ng/uL (0.36 s exposure). For the a-strand of either H4 or H2B probe (H4-a and H2B-a), a shifted complex A is observed only with the addition of a higher concentration of the gTBP protein. For the b-strand of either H4 or H2B probe (H4-b and H2B-b), a shifted complex B1 is observed at a lower concentration of gTBP protein. The B1 shifted complex transitions to a higher shifted complex (B2) with increasing concentrations of gTBP with the b-strand probes. The B1 band potentially represents monomeric binding between gTBP and the DNA probe, and the B2 band potentially represents multimeric binding with two or more bound gTBP proteins to the DNA. *B*, the average relative proportion of each band seen in the corresponding EMSA (n = 4). Each band corresponds to a free DNA probe or a form of gTBP-DNA complex. In the case of the H2B-a graph, the additional band may be due to small amounts of the DNA forming a secondary structure which has a slightly slower electrophoretic motility *versus* the unfolded ssDNA. Error bars represent standard deviation. *C*, the total percentage of bound DNA with increasing amounts of gTBP for the corresponding DNA probe, as determined using the curve for the free probe in B. The curves show nonlinear regression to the Hill-Langmuir equation for binding reactions at equilibrium. The regression was done using two degrees of freedom: the equilibrium dissociation constant (K_D_), and the Hill coefficient (n); both of which are shown on each graph.

By titrating gTBP in EMSA experiments, we were able to observe multiple bands corresponding to gTBP-DNA complexes that are dependent on both the ssDNA probes used and the concentration of gTBP (Fig. 6*A*). For H4-b and H2B-b, we observe both a lower shifted band (B1 complex) and a higher-shifted band (B2 complex) that appears at higher protein concentrations. Since native PAGE analysis suggests that the recombinant GST-gTBP used in our assay forms dimers and higher multimers (Fig. S6), we hypothesize that the B1 complex represents binding involving one gTBP dimer, with each monomer binding one ssDNA molecule. The B2 complex likely represents the binding of additional molecules of gTBP to the ssDNA probe as the proportion of B2 complex increases at higher gTBP concentration while the proportion of B1 complex decreases (Fig. 6*A*). Only a high migration band (A complex) is seen for H4-a and H2B-a at high gTBP concentrations (Fig. 6); there does not appear to be a band corresponding to a lower complex at either low or high concentrations of gTBP. Potentially, the labeled ssDNA a-strand probes bind to an already formed multimer of gTBP. Since there does not seem to be intermediate bands corresponding to the binding of lower gTBP oligomers including a dimer for the H4-a and H2B-a probes, formation of the A complex on the a-strand probes may proceed in a different binding manner than either B1 or B2 complexes observed with the b-strand probes.

We estimated the equilibrium dissociation constants (K_D_) for the four ssDNA probes used in our study (Fig. 6*C*). Since the stoichiometric proportions of the gTBP-ssDNA for the B1 and B2 complexes are unknown, we are unable to accurately determine K_D_ values for these complexes separately. Therefore, we used the relative intensity of unbound ssDNA probe to determine estimated K_D_ values for total binding as a function of protein concentration for both the A complex formation and combined B1 and B2 complex formation (or simply the B complex in total). Using nonlinear regression to the Hill equation, we show that equilibrium binding constants (mean ± SEM) for H4-a (500 μM ± 50) and H2B-a (7200 μM ± 2500) are significantly greater than that of either H4-b (7.00 μM ± 0.2) or H2B-b (5.0 μM ± 0.2) (n = 4 for each; *p* < 0.001). We also note that the Hill coefficient (n) for total binding with both H4-b and H2B-b probes is ∼ 2, potentially indicating positive cooperativity in the transition from the B1 to the B2 complex. This suggests that the binding of the first gTBP facilitates the binding of additional gTBP molecules to the b-strand probes.

**Figure 7:**
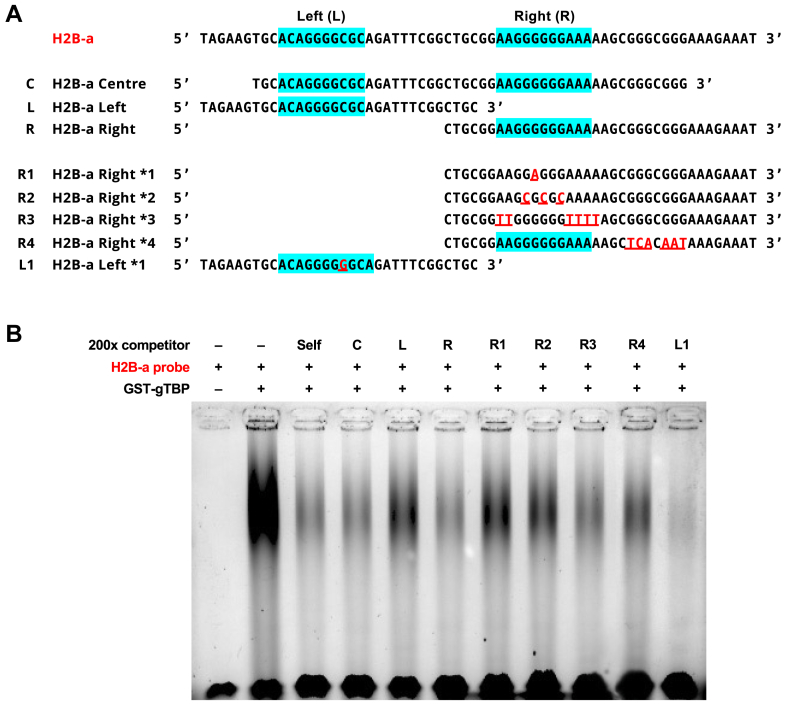
***Giardia* TBP binds in a sequence-dependent manner to the DNA motif identified from a high-throughput protein binding microarray.***A*, sequences of DNA probes and competitors used. *Blue* highlighted regions indicate matches to the DNA motif determined *via* the microarray. *Red* and underlined letters in mutant competitors indicate nucleotides modified from the parent sequence. *B*, EMSA analysis with labeled H2B-a DNA probe against various subregion and mutant competitors (120 s exposure). Representative EMSA of four replicates.

To analyze the sequence requirements for the gTBP binding to DNA in the A complex of the a-strand probes, we modified H2B-a in EMSAs (Fig. 7). The contribution of each of the PBM matches in H2B-a (labeled as left and right) was examined by using subregions of the sequence as unlabeled competitors against the full-length sequence. The results showed that the right motif is a better competitor than the left one. When mutations were made to the central 6 G nucleotides in the right motif (R1 and R2), the ability of this subregion to compete decreased. When we introduced a single nucleotide change in the left motif to increase the central G nucleotide stretch from four to six, the mutated sequence (L1) became a better competitor. These results indicate that one factor in the specificity of gTBP binding in the A mode is the number of consecutive guanine nucleotides in the sequence. However, the nucleotides flanking the central guanines within the right motif have only a minor contribution to gTBP binding since the R3 mutant still competed. We noted the presence of G-rich “islands” downstream of the right motif, which could contribute to the potential formation of secondary structures known as G-quadruplexes (29). Mutations made to these downstream G-rich regions (R4) resulted in a slight decrease of competition against the full-length probe for binding to gTBP compared to the sample where the unmutated subregion (R) was used as the competitor.

**Figure 8:**
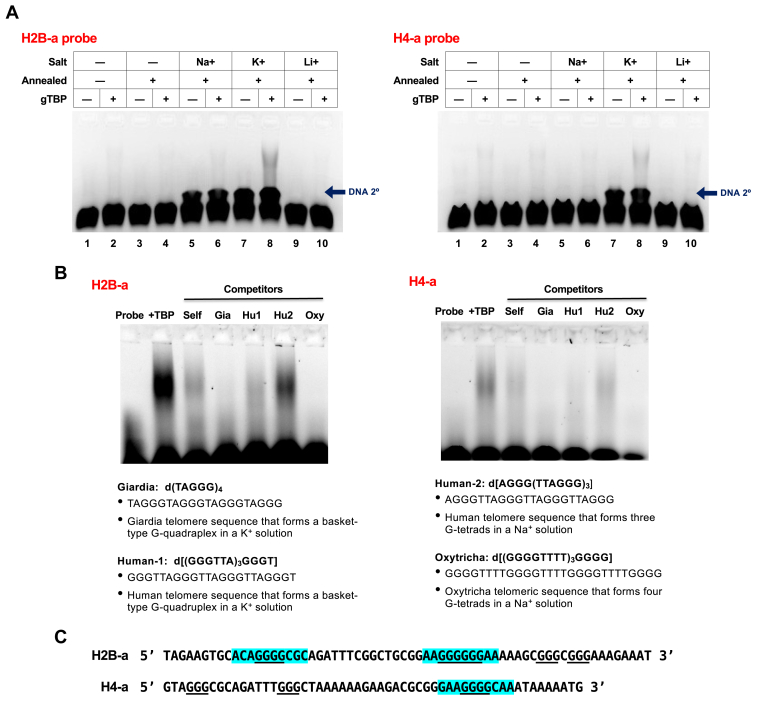
**TheAmode of binding may also involve the recognition of DNA secondary structures by gTBP**. *A*, the labeled H2B-a and H4-a ssDNA probes were each heated and then cooled slowly to promote secondary structure formation (Annealed +) in the presence of either Na^+^, K^+^, or Li^+^ as cations before the addition of gTBP (6 s exposure). This exposure time was selected to observe the separation of the two bands at the *bottom* of the gel. The *blue arrow* indicates the extra band (DNA 2°) at the *bottom* of the gels that likely represents the formation of DNA secondary structures in the probe in some samples. *B*, the labeled H2b-a and H4-a DNA probes and unlabeled DNA sequences representing telomeric repeats from *Giardia* (Gia), human (Hu1 and Hu2), and Oxytricha (Oxy) were each heated and then cooled slowly to promote secondary structure formation (Annealed +) in a buffer containing Na^+^ and K^+^. The annealed, unlabeled telomere samples were used as competitors for gTBP binding to the labeled H2B-a and H4-a probes (30 s exposure). *C*, sequences of the DNA probes used with the PBM motif are highlighted in *blue* and polyG regions are underlined.

**Figure 9:**
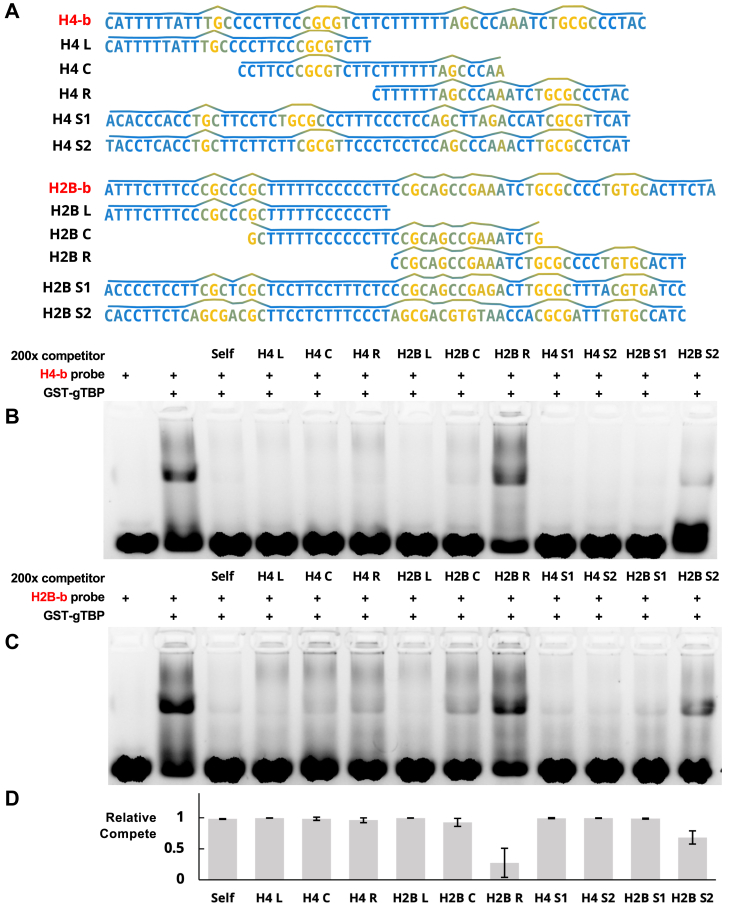
***Giardia*****TBPbinds single stranded DNA sequences with similar base stacking energy profiles**. *A*, sequences and base stacking energy profiles for DNA probes and competitors used. *Blue* regions indicate low base stacking energy and *yellow* regions indicate high base stacking energy (see Table 1). The energy profile is also indicated by the relative level of the line directly above each sequence. *B*, representative EMSA (n = 2) with labelled H4-b DNA probes against unlabelled competitors (2.3 s exposure). “L,” “C,” and “R” competitors correspond to the *left, centre*, and *right* subregions of their respective parent strand. “H4-similar” competitors ("S1” and “S2”) were generated *de novo* to be similar in base stacking energy profile to H4-b, but as different in their sequences as possible. *C*, similar to (*B*), using H2B-b labelled probes. *D*, quantification of binding to competitors on EMSAs for both DNA probes (n = 2 each; n = 4 in total). Relative strength of competition is determined with respect to the lane containing only DNA probe and gTBP. Relative binding value ranges from 0 (no competition/low binding affinity) to 1 (complete competition/high relative binding affinity). Error bars represent standard deviation.

To further explore the possibility that DNA secondary structures could contribute to gTBP binding, we modified our EMSAs to promote secondary structure formation in the H2B-a and H4-a probes. Here, probes were denatured using heat and renatured through slow cooling, in the presence of different monovalent cations, before incubating with gTBP (Fig. 8*A*). An additional, slower migrating band (see blue arrow)—slightly above the band representing the unbound linear probe—is observed in the annealed Na^+^ and K^+^ samples with the H2B-a probe (lanes 5–8 in left gel) and in the annealed K^+^ sample containing the H4-a probe (lanes 7 and 8 in right gel). This observation is consistent with DNA secondary structure formation in the probes in these reactions. When potassium ions (K^+^) are added to the samples, we observed an enhancement of gTBP binding to the H2B-a probe (see Lane 8 vs. Lane 2 in left gel). These same conditions also increased gTBP binding to the H4-a probe (see Lane 8 vs. Lane 2 in right gel) although no increases in binding were observed for the H2B-b and H4-b probes (data not shown). Since G-quadruplexes formed at telomeres are well characterized, we used telomere sequences from *Giardia*, humans (Human-1 and Human-2), and a ciliated protist *Oxytricha* (30) as competitors in EMSAs with the H2B-a and H4-a probes (Fig. 8*B*). The *Giardia*, Human-1 and *Oxytricha* sequences were strong competitors. We also observed that polyG is a very effective competitor for binding to the a-strands of H2B and H4 but not homopolymers of A, C, or T (Fig. S10*A*) and that the b-strands of H4 and H2B are effective competitors for gTBP binding to H4 and H2B a-strands (Fig. S10*C*). For the latter, since complementary base pairing between the a- and b-strands would produce dsDNA with a concomitant reduction of ssDNA probes to bind gTBP, we cross-competed the H4-a probe with the H2B b-strand and, similarly, cross-competed the H2B-a probe with the H4-b strand. Other competitors used are the *de novo* generated b-strand sequences that match the base stacking profiles of the parental H4-b and H2B-b but are as dissimilar as possible to the parental sequences that will be further described in Figure 9.

### *Giardia* TBP-DNA B complexes may form using structural features of single stranded DNA

Since we were unable to determine a consensus DNA sequence for gTBP binding in the B binding mode, and the preceding experiments suggested that DNA secondary structures can impact gTBP binding in the A-mode, we expanded our analysis to include the structural features of ssDNA in the context of gTBP binding in the B-mode. Single-stranded DNA is inherently more flexible than dsDNA, and one major determinant of ssDNA flexibility is base stacking energy, which is the energy of the aromatic interactions of neighboring bases along the strand of DNA (10, 11). A highly stacked region of ssDNA would be compact and rigid, while a region with a less stable base stacking would be more elongated and flexible. A measurement of the extent of base stacking is the energy potential for each adjacent pair of bases (U_S_). To simplify our following discussions, here we use the term high-base stacking to represent a high negative energy (U_s_ ∼ −4.9 to −5.09 kcal/mol), or rigid sections, and low-base stacking to represent low negative energy (U_s_ ∼ −4.2 to −4.15 kcal/mol), or flexible sections. See Table 1 for the U_s_ values for each dinucleotide.

**Table 1:** Base stacking energy values for all single-stranded dinucleotides using the model developed by Chakraborty, *et al.* (51)

ssDNT	U_s_	*h* (kcal/mol)	s	T_m_ (K)
CT	−4.15	4.13	0.71	288.3
CC	−4.17	4.15	0.98	288.3
TT	−4.19	4.17	0.89	288.3
TC	−4.20	4.18	0.94	288.3
AT	−4.21	4.18	0.87	293.0
AC	−4.24	4.21	0.92	293.0
CA	−4.27	4.24	0.79	293.0
TA	−4.30	4.28	0.65	293.0
AA	−4.75	4.67	0.94	322.0
GT	−4.89	4.70	1.69	332.6
GA	−4.93	4.82	0.98	333.6
AG	−4.95	4.83	1.06	333.6
GC	−4.99	4.73	2.41	331.9
TG	−5.05	4.86	1.73	332.6
CG	−5.06	4.81	2.33	331.9
GG	−5.09	5.13	−0.29	353.9

Intermediate values for adjustable parameters relating to the enthalpy (*h*) and entropy (*s*) of stacking interactions, and the melting temperature of the dinucleotide (*T*_*m*_) are shown.

First, we generated per-nucleotide base stacking energy profiles of the H4-b and H2B-b sequences as described in the Materials and Methods (visual representations by line above each sequence in Figures 8*A* and 9*A*; raw data in File S2). Next, we tested the relative ability of different sections (left, center, and right) of the b-strands of the H4 and H2B promoters to compete with the full-length probe for gTBP binding. We noted that certain base stacking factors may influence gTBP binding (Fig. 9). Sections of either H4-b or H2B-b which contain long stretches of C or T, which are punctuated by G nucleotides (H4 L, H4 C, H4 R, and H2B L), are better competitors than sections with fewer of these features (H2B C), or none of them (H2B R) (Fig. 9). The sequences that competed best seem to correlate to sequences with low base stacking that are punctuated with a few points of high base stacking. However, the uninterrupted length of the low base stacking region (LBSR) also appears to be one factor for gTBP binding. For example, H2B-R, which is the sequence that competed the least, is approximately the same total length as H2B-C, but H2B-R is missing the long uninterrupted LBSR (shown in blue) in H2B-C. We propose the pronunciation of LBSR as “LOBSTER” for *LO*w *B*ase *STa*cking *E*nergy *R*egion.

Next, we used a custom Python script to generate sequences that are similar to the parental sequences (H4-b, H2B-b) in terms of their base stacking energy profile (see lines above each sequence) but are as dissimilar as possible in terms of their actual sequence. We found that all four generated sequences—H4 S1, H4 S2, H2B S1, and H2B S2—can compete effectively against their parental counterparts in EMSAs (Fig. 9). Since base stacking energy is dependent on sequence, the generated sequences are not wholly different. For instance, since the energy of dinucleotides such as CT, CC, TT, *etc.* are similar (see Table 1), regions in the parental strand which are CT-rich tend to also be CT-rich regions in the generated strands, even if the exact sequences are different. Interestingly, H2B S2, which does not compete as well as H2B S1, is more *dissimilar* in its base stacking energy profile to the H2B parental sequence in the left (L) region (see the energy profile of “CGCCCGC” sequence in yellow in H2B-b in Fig. 9*A*). In both H2B and H2B S1, the base stacking energy profile of this region has two high energy peaks separated by a steep valley created by the low energy nucleotides CCC or CTC. In contrast, the equivalent region in H2B S2 has a single broad high energy peak with a much shallower valley created by the nucleotides AC. These results are consistent with the idea that properties deriving from base stacking energy (*e.g.* ssDNA flexibility, areas for ssDNA-protein stacking interactions) can impact binding gTBP to ssDNA.

### Determining a minimum binding sequence for the B-mode of gTBP

**Figure 10:**
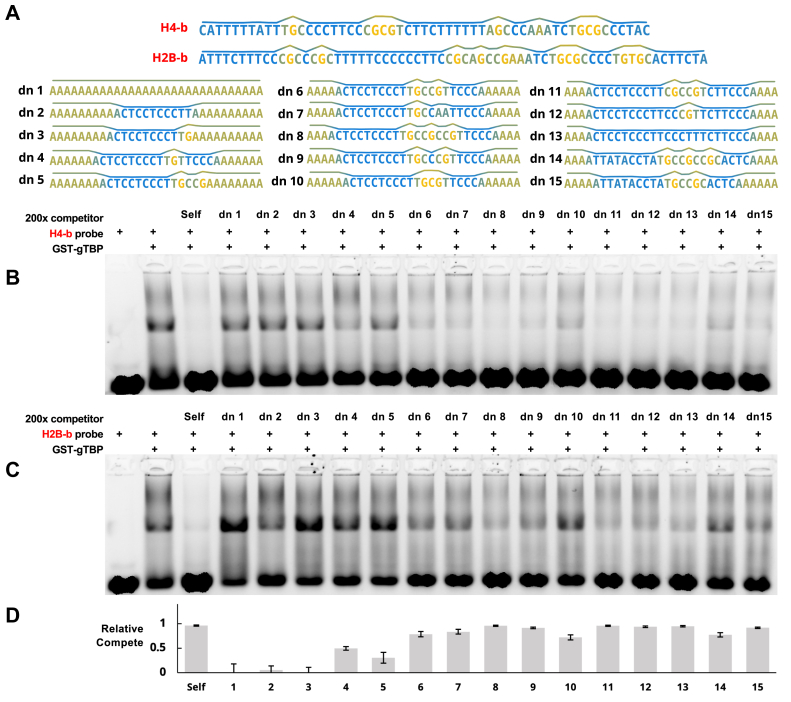
***Giardia*****TBPdifferentially binds to a series of *de novo* competitor sequences with incremental changes to assess the minimum binding sequence.***A*, the sequence and base stacking energy profiles (line plot above each sequence) are shown for the H4-b and H2B-b probes and the set of DNA competitor sequences. *Blue* regions indicate low base stacking (Us less than approx. −4.8) and yellow regions indicate high base stacking (Us greater than approx. −4.5). *B*, representative EMSA (1 of 2) with labelled H4-b DNA probes against unlabelled competitor sequences. *C*, similar to *B*, using H2B-b-labeled probes. *D*, quantification of binding to competitors on EMSAs for both DNA probes (n = 2 each; n = 4 in total). Relative strength of competition is determined with respect to the lane containing only the DNA probe and gTBP. Relative binding value ranges from 0 (no competition/low relative binding affinity) to 1 (complete competition/high relative binding affinity). Error bars represent standard deviation.

With partial understanding of how gTBP binds the subsections and derived sequences of H4-b and H2B-b, we sought to determine the minimum requirements for binding in the B mode using completely *de novo* competitors (Fig. 10). We started with a polyadenine sequence, which we have shown does not bind gTBP (Fig. 4, lane labelled "polyA" and labelled as dn 1 in Fig. 10), as the base for building an effective competitor for gTBP binding. We made a series of competitors with incremental modifications to this base sequence that were designed to primarily affect its base stacking energy profile. We modified the lengths and number of LBSRs (blue font), as well as the disruption of these regions by adding nucleotides (yellow font) that create high energy points (HEPs) as shown in Figure 10*A*. We define HEPs as isolated G or AA nucleotides, corresponding to the dinucleotides of highest base stacking energy (*i.e.* GN, NG, or AA; as seen in Table 1). These parameters were chosen based on our observations from what appeared to bind well in Figure 9.

We observed no competition for dn 1 containing the polyA sequence as expected (Fig. 10). Poor competition was observed for dn 2, dn 3, and dn 5, which have the shortest uninterrupted LBSR lengths among these samples (dn 2 has 10 nt, dn 3 has 9 nt, and dn 5 has 9 nt). In comparison, dn 13, which has an uninterrupted LBSR of 22 nt, competes well for binding to gTBP. The other sequences varied in the number of disrupting nucleotides from 3 nt in dn 12 (CGT) to 9 nt in dn 14 (TGCCGCCGC) and dn 15 (TTATACCTA), but have approximately the same ability to bind gTBP. These interrupting regions are composed of varying numbers of HEPs.

Overall, we can deduce two general features for binding between gTBP and ssDNA in the B-mode.

(1) **Binding occurs with sequences containing an uninterrupted LBSR greater than ten nucleotides.** gTBP binds well to polyC, polyT (Fig. 4), H4-b, H2B-b, and dn 13 but not to dn2 (Fig. 10). Although these sequences all contain long stretches of CT nucleotides, we show that binding does not depend on specific sequences but rather is associated with regions of low base stacking energy that are longer than 10 nucleotides.
(2) **Binding occurs with longer LBSR sequences containing the insertion of up to nine nucleotides containing varying numbers of HEPs.** A LBSR ranging from 19 to 22 nt (dn 6–15 in Fig. 10) can tolerate interruptions of up to nine nt containing up to three HEPs (dn 8 and dn 14) without affecting its ability to compete for gTBP binding.

We sought to determine a more definitive pattern for binding in this B mode by utilizing our existing dataset of semi-quantified values for the relative binding affinity for ssDNA sequences to develop a regression model as detailed in Fig. S7. However, our final trained model yielded parameter weights and bias that only moderately predict the relative binding affinity of sequences used in training (R² = 0.80). We then tested sequences corresponding to promoters from 12 randomly selected *Giardia* genes in EMSAs with gTBP (Fig. S8, sequences listed in File S2), and observed that the model does not accurately predict the binding affinity of these sequences (R² = 0.15) (Fig. S9).

**Figure 11:**
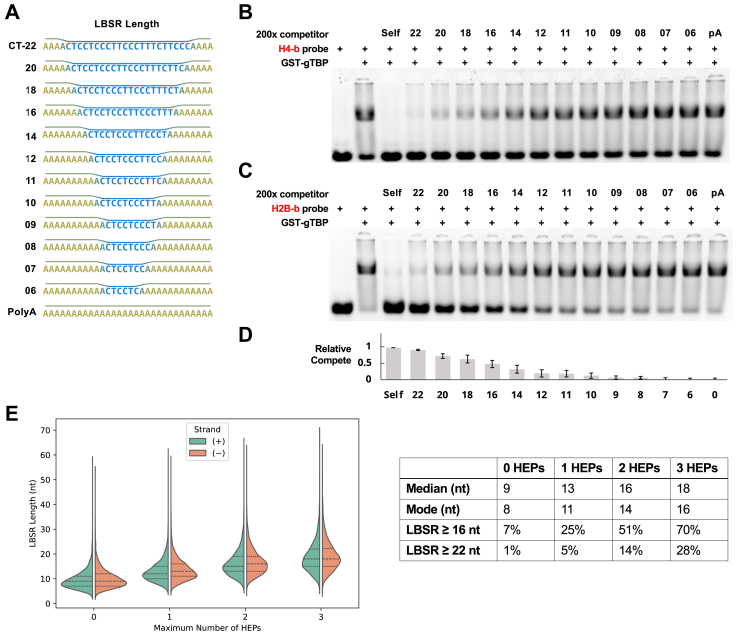
**The effect of LBSR length on gTBP binding and frequency of LBSR in *Giardia* gene promoters.***A–D*, the effect of low base stacking energy region (LBSR) length on gTBP is analyzed by testing sequences with decreasing lengths of LBSR as competitors to the H4-b or H2B-b probe (1.3 s exposure). *E*, split violin plot showing the distribution of different lengths of LBSRs in the promoters of *Giardia* genes with a maximum of 0 to three high energy points (HEPs) permitted within the LBSR. Long dashed lines indicate median values, and short dashed lines indicate first and third quartiles. The table shows the median, mode, and the percentage of genes that have LBSR lengths ≥ 16 nt and ≥ 22 nt in their promoter for each maximum number of HEPs permitted.

Since the regression modeling was unsuccessful, we sought to better define gTBP binding requirements by expanding our analysis of the impact of LBSR length on gTBP binding. To do this, we took the 22 nt LBSR region of dn13 shown in Figure 10 that competes well for gTBP binding and gradually reduced its length within a base sequence of polyA (Fig. 11*A*). A direct correlation between the length of the LBSR and its ability to compete for gTBP binding was observed (Fig. 11, *B*–*D*). A sequence containing a 10 nt LBSR is a poor competitor and agrees with the poor competition observed for dn13 that also has a 10 nt LBSR in Figure 10. In comparison, a 16 nt LBSR competes at ∼50% relative to the self-competitor (Fig. 11*D*).

Next, we surveyed the promoters of the genes in GiardiaDB for the occurrence of LBSRs with increasing numbers of HEPs (Fig. 11*E* and File S6). We conservatively limited the number of HEPs from 0 to 3. The distribution of gene promoters from each search criterion increased in variance and central tendency (*i.e.* median, and mode) with increasing number of HEPs allowed (Fig. 11*E* and File S6). Since an uninterrupted LBSR length of 16 nt gave 50% competition in Figure 11*D*, we determined the percentage of genes that contain this length or longer in their predicted promoters. We observed that 7% of *Giardia* genes have an uninterrupted LBSR equal to or greater than 16 nt, whereas 70% of genes have this LBSR length if up to three HEPs are permitted. If we use a 22 nt LBSR as the cutoff, then only 1% of the genes have this uninterrupted length in their promoter, but this increases to 28% of the genes if up to three HEPs are permitted. Thus, using the “rules” uncovered by our *in vitro* gTBP-binding studies, we identified potential promoter binding sites in the genome of *Giardia*.

## Discussion

*G. intestinalis* is an example of genetic reductionism with a relatively small genome (12.08 Mb) containing short separation between genes and loss of introns within genes (7). *Giardia* orthologs of components in essential pathways such as initiation of DNA replication and polyadenylation of mRNA appear to be absent (7). The promoters of *Giardia* protein-encoding genes are also short (50–60 bp), mostly TATA-less, and have an AT-rich rather than a CT-rich initiator element found in other eukaryotes (21, 22, 28). The *Giardia* RNA polymerase II is resistant to 1 mg/ml α-amanitin, and it lacks the heptapeptide repeat in its C-terminus (31). The components of the basal transcription factors required for the assembly of an RNA pol II pre-initiation complex in *Giardia* are also unknown (5). *Giardia* has an ortholog of TBP (gTBP), yet it is among the most highly divergent among all other known TBPs, missing three of the four key phenylalanines that intercalate dsDNA on each side of the TATA-box, as well as substitutions in 10 out of the 11 amino acids that are implicated for DNA recognition and specificity (Fig. 1*A*) (5, 13). Despite the divergence of the gTBP amino acid sequence, its predicted structure resembles that of other eukaryotic TBPs (Fig. 1*B*) with a saddle shape and a stirrup extending from each side of the saddle. Additionally, we identify nuclear localization of gTBP which provides support for a role in transcription (Fig. 2, *A* and *C*).

The cross-sectional diameter and dyad symmetry of dsDNA is matched in the DNA-binding site of the most well-known TBPs by a saddle-shaped structure with dyad symmetry in the fold as well as in most of the amino acid side chains that contact the minor groove of the DNA double helix. While gTBP retains the dyad symmetry of the protein fold, few residues have identical symmetry-matched partners in the DNA binding site, which is also narrower. Together these features may explain the preference of gTBP for binding single-stranded rather than double-stranded DNA.

AlphaFold3 analysis of gTBP and hTBP with ds and ssDNA as potential ligands indicates that gTBP could only accommodate ssDNA whereas hTBP could accommodate either (Fig. S3). The latter finding for hTBP has been validated experimentally (32, 33). Transcription factor binding to ssDNA can have varied functions. For example, ssDNA binding by hTBP “traps” the active promoter in its open complex which is necessary for transcriptional initiation (32). Another proposed function of hTBP is that it may have similar roles as the single-stranded DNA-binding protein (SSBP) in DNA replication such as to prevent ssDNA from reannealing, protect ssDNA from degradation, and to interact with other proteins (32). Preferential binding of ssDNA can also be important for gene specific transcriptional regulation as observed during FUSE-binding protein (FBP) mediated transcription of human c-Myc (34) and PBF-2 mediated transcription of pathogen related genes in plants (35).

Our analysis using AlphaFold3 showed that ssDNA oligonucleotides of lengths 8 to 15 nt could be docked to the expected DNA-binding pocket of both gTBP and hTBP, although these models varied in the conformational flexibility and orientation of the DNA within the pocket and showed little dependence on sequence. Interestingly the structure of gTBP showed more flexibility than hTBP, notably a hinge motion between the two halves of the protein, whereas the structure of hTBP is more rigid and did not differ significantly among model structures. We speculate that the increased flexibility in gTBP may allow it to bind a greater diversity of ssDNA than hTBP. In typical TBPs, such as those from human or yeast, that bind dsDNA, the four phenylalanines in the underside of the saddle induce a kink on each side of the dsDNA binding site to help unwind the DNA (13). Notably, gTBP lacks three out of the four phenylalanines and its ssDNA binding activity is likely preceded by dsDNA unwinding *via* a TBP-associated factor (TAF) with helicase activity *in vivo*.

AT-rich sequences containing the transcription start sites function as transcription initiator elements in *Giardia* promoters (21, 22, 28). Since *Giardia* gene promoters lack TATA-boxes, it was assumed that gTBP would bind the AT-rich initiator (7). However, when we tested gTBP’s ability to bind sequences corresponding to different *Giardia* promoters in EMSAs, we did not observe a consistent correlation among DNA sequences that could bind gTBP and the presence of AT-rich initiators, TATA-box like sequences, or other AT-rich regions (Fig. 4). Furthermore, poly(C) and poly(T) sequences can compete for binding to gTBP, but poly(A) and poly(G) sequences cannot (Fig. 4). Interestingly, hTBP showed similar preferences for binding ssDNA containing poly(C) and poly (T) (32, 33). Our analysis of gTBP’s binding to CT-rich sequences suggest that it is the flexibility of these ssDNA sequences that is involved in this specificity. In our study, gTBP binding to CT-rich sequences and other sequences with inherent flexibility is referred to as the B mode (Figs. 3, 4, 6, 8 and 9). We also discovered another binding mode of gTBP in our PBM and EMSA results that we referred to as the A mode (Figs. 3 and Figure 5, Figure 6, Figure 7, Figure 8) that involves the binding of poly(G) tracts. Each of these modes of binding will be discussed in more detail below.

The dissociation constants for both modes of gTBP binding were obtained by the analysis of EMSA results (Fig. 6). The dissociation constant for the B mode observed for the b-probes (H4-b and H2B-b) is 5 to 7 μM. This is considerably weaker binding compared to the binding of yeast TBP (KD 0.3–2 nM) (36) and human TBP (0.5 nM) (37) toward their double-stranded TATA sequences. Even compared to nonspecific binding of single-stranded phage M13 DNA to yeast TBP (KD 20 nM) (36), the binding of gTBP to its preferred single-stranded targets is still 350-fold weaker. On the other hand, gTBP binding to its preferred ssDNA in the B mode exhibits positive cooperativity with a Hill coefficient of 2, which indicates that binding of the first gTBP facilitates the binding of additional gTBPs to the probe. This is consistent with the EMSA titrations which show a diminishment in the intensity of the lower band (B1 complex) and the formation of a higher band (B2 complex) for the H4-b and H2B-b single-stranded probes which would be expected for a probe that binds more than one gTBP (Fig. 6*A*). In contrast, the binding data for human TBP to dsTATA probe was adequately fitted to a hyperbolic binding function with 1:1 binding of protein to DNA. The structural changes that accompany cooperation are unknown. One possibility is that the binding of the first gTBP induces change in the conformation of the single-stranded probe which promotes binding of the second gTBP. Binding of this second gTBP may involve interactions with DNA alone or with both the DNA and the first bound gTBP. It is unlikely to be solely a protein-protein interaction; while human TBP (38) and yeast TBP (39) form dimers and oligomers respectively, they do so only in the absence of DNA.

The weak binding of gTBP we observed in our EMSAs suggest that other proteins or co-factors that are currently unidentified are likely required for gTBP binding within the cell. In other eukaryotes, TBP functions within a TFIID complex alongside other proteins. In *Giardia*, it is probable that one of these additional proteins possesses helicase activity to unwind dsDNA into ssDNA to facilitate gTBP binding, while other proteins in the complex could stabilize gTBP-ssDNA interactions.

We do not consider the pathway for the formation of B2 complex observed with the b-probes the same as the A complex observed with the a-probes although both complexes are observed with higher gTBP concentrations (Fig. 6*A*). The B mode seems to consist of gTBP binding to DNA which is correlated with a low base stacking energy (U_s_ ∼ −4.2 kcal/mol) and greater flexibility (Figs. 8 and 9). Furthermore, the B2 complex is a transition from the monomeric gTBP in the B1 complex to form oligomeric gTBP on the DNA through cooperative binding with increasing protein concentration. In contrast, the A complex involves the recognition of G-tracts in ssDNA and there is no transition to the formation of this complex from a complex containing lower oligomers or monomers of gTBP (Fig. 6). The dissociation constant for the A binding mode observed for the a-probes (H4-b and H2B-b) is 500 to 7200 μM, which is much higher than for the B mode. One reason for the weak binding in the A mode may be that it requires the formation of a G4 structure on the DNA, and our EMSA conditions did not favor the formation of this structure.

The A-binding mode was detected in the PBM analysis (Fig. 5) and in the EMSAs when a high concentration of gTBP was used with the H4 and H2B a-strand probes (Fig. 6). This mode is sequence-specific and is dependent on a core sequence containing 4 to 6 consecutive guanine nucleotides (Fig. 7). Single-stranded DNA sequences containing runs of three or more guanine nucleotides can form secondary structures called G-quadruplexes or G4s (29) that have roles in gene regulation, telomere functions, DNA replication, and genome stability (40). G4s are stacked tetrads of guanine nucleotides that are associated with Hoogsteen base pairing to form a ring. Different families and subtypes of G4s can form, but one type is from the intramolecular interactions of four isolated “islands” of guanines with a consensus sequence of GaNbGaNbGaNbGa (a = 2–5, b = 1–7) (41). We identified a potential candidate G4 sequence in the H2B-a sequence that binds gTBP. A mutant of H2B-a lacking the two downstream guanine islands was a less effective competitor for gTBP binding in EMSAs (R4 in Fig. 7). This prompted us to examine the effect of secondary structure formation of the H2B-a probe on gTBP binding.

We observed that conditions that favor DNA secondary structures resulted in increased gTBP binding (compare lane 8 to lane 2 in gels shown Fig. 8*A*). Interestingly, this enhanced binding is only observed when K^+^ is in the annealing buffer but not when it is replaced by either Na^+^ or Li^+^ (compare lane 8 to lanes 6 and 10 in gels shown in Fig. 8*A*). G4 structures are stabilized by a central cation which interacts with the lone electron pairs on the center-facing oxygens, with the order of monovalent cations providing the most stability as K^+^ > Na^+^ > Li^+^ (42). We also observed an enhancement of binding to the H4-a probe in the presence of K^+^ despite its lack of the downstream Gs observed in H2B-a. However, the H4-a sequence has the PBM motif with the four central Gs as well as two other regions with 3 Gs in a row (see sequences underlined in Fig. 8*C*) that may form a non-canonical type of G4. Notably, the *Giardia* telomere consists of repeats of TAGGG that contain only 3 Gs (43, 44) and DNA with four repeats of this sequence can form two subtypes of intramolecular G-quadruplexes (30). We used sequences with the *Giardia* telomere repeat as well as other telomeric repeats from human and another protist in EMSAs with the H2B-a and H4-a probes and found that they all competed for protein binding except for the human telomere sequence (Hu2) that forms three G-tetrads stabilized by Na^+^ (Fig. 8*B*). This indicates specificity for the type of G4 that binds to gTBP. Overall, our observations that K^+^ selectively enhances gTBP binding to the annealed probes, and specific telomere sequences are able compete for this binding, support the possibility of G4 formation in the two promoter sequences.

G4s can both repress and activate transcription through several distinct mechanisms (45). For example, stabilization of G4 structures in the human c-Myc promoter results in steric hindrance to transcription initiation and elongation (46). In contrast, G4 stabilization of R-loop formation enhances transcription (47). During oxidative stress, damage to the guanines in G4 structures activates DNA repair that results in apurinic sites that recruit the binding of transcription factors and enhanced transcription (48). The result is a set of genes that are upregulated by DNA damage mediated through a G4 complex. Finally, G4s are prevalent in human gene promoters, and many serve as binding sites for transcription factors (49).

To examine whether the B mode of gTBP binding to DNA is dependent on the DNA structure, we used the base stacking interactions of adjacent nucleobases in ssDNA sequences as a proxy measure for its overall flexibility. Structures formed with ssDNA are inherently more flexible than those formed from dsDNA due to the absence of stable Watson-Crick base pairing (10, 50). Chakraborty and coworkers (51) have developed a coarse-grained computational model that accurately models ssDNA flexibility using a sum of component forces, including bond stretch, bond angle, excluded volume, electrostatic, and single-stranded stacking interactions. Of these component forces, the stacking interactions between the aromatic bases on adjacent nucleotides (U_S_) has a particularly strong impact on the local flexibility within different regions on ssDNA sequences (10, 11). Groups of adjacent nucleotides can transition between rigid stacked configurations and more flexible unstacked configurations, exposing the nucleobases to permit π-π stacking interactions with aromatic side chains on proteins. These two linked factors—ssDNA flexibility and the stacking interaction between ssDNA and proteins—have been shown to modulate specificity within binding interactions between ssDNA and proteins (52). Therefore, using the base stacking energy profile of a given ssDNA sequence allows for a simple proxy measure of ssDNA flexibility and propensity for ssDNA-protein interactions.

The B1 gTBP-ssDNA complex most likely involves a monomer of gTBP binding to ssDNA. Since the gTBP used in this study is a GST fusion protein, it forms a dimer *via* its GST domain (Fig. S6). However, since structural analysis of other TBPs bound to DNA show it binding as a monomer to one DNA molecule (3, 53), it is likely that the band corresponding to the B1 complex we observed in this study is a result of a GST-gTBP dimer with each subunit or monomer binding to its own molecule of DNA. This B mode is dependent on DNA structure where one of its parameters is its flexibility as measured by base-stacking energy of adjacent nucleotides (Table 1). Our results show that gTBP prefers binding to regions of low base stacking energy (U_s_ ∼ −4.2 to −4.15 kcal/mol) that correspond to weak base-stacking and more DNA flexibility (Figs. 9 and 10). Regions of low base-stacking energy also correspond to regions that are CT-rich. Notably, we also observed that polyC and polyT sequences are very effective competitors for gTBP binding (Fig. 4). Similar to our observations in gTBP, hTBP has a stronger affinity toward polyC or polyT ssDNA sequences compared to single- or double-stranded TATA-box elements (32, 33).

Having a TBP which binds ssDNA in addition to or in place of traditional TATA-box elements implies unique functional relevancy in transcription in *Giardia*. In their study on human TBP-ssDNA interactions, Irani *et al.* (32) describe several possible biological roles including that (a) TBP may partially unwind dsDNA when it kinks DNA at the TATA-box, then bind to one strand, stabilizing an open complex; (b) TBP may be involved in both transcription and DNA replication; and (c) TBP may bind to the CT-rich initiator (Inr) motifs on TATA-less promoters. In addition to these possibilities, the unusual DNA specificity of gTBP may help to explain *Giardia*-specific phenomena. *Giardia* is known to undergo bidirectional transcription originating from a single promoter site, often producing sterile transcripts which do not code for any protein (54). Degenerate AT-rich TATA and Inr elements have been implicated in allowing for this bidirectionality (55), but our results show that gTBP prefers binding to CT-rich sequences in the B-mode rather than to AT-rich sequences such as the initiator in *Giardia* promoters. The diverse forms of ssDNA binding outlined here may potentially impact bidirectional transcription.

A search of the *Giardia* genome identified LBSRs in the promoters of genes encoding proteins, tRNAs, and rRNAs (File S6). Our EMSAs showed that a LBSR length of 16 nt resulted in 50% reduction of the gTBP binding compared to a 22 nt LBSR (Fig. 11*D*). If this length is used as a cutoff, we observed 7% of *Giardia* gene promoters have an uninterrupted LBSR equal or greater than this length, but this increases to 70% if up to three HEPs are allowed (Fig. 11*E*). The results in Figure 10 showed that a LBSR of 22 nt (dn 8 and 14) each containing three HEPs can bind gTBP. If we use a 22 nt LBSR as the cutoff, then only 1% of the *Giardia* genes have this uninterrupted length in their promoter but this increases to 28% if up to three HEPs are permitted. Since TBP in other eukaryotes is required for transcription of genes, even if it does not bind directly to all promoters (6), the 22 nt LBSR with up to three HEPs is a more likely model for the frequency and distribution of the B-mode binding sites of gTBP in *Giardia* gene promoters.

Our work provides evidence for the ability of TBP from *Giardia* to bind single-stranded DNA in a manner not conventionally expected of TATA-box binding proteins and suggests that structural properties of ssDNA such as flexibility—mediated by and measured through base stacking energy—may influence this interaction. In another binding mode, higher oligomers of gTBP may bind and stabilize DNA secondary structures such as G-quadruplexes. Consequently, gTBP may play multiple, context-dependent roles in *Giardia* transcription. These findings expand our current understanding of transcription, provide a fresh perspective on the role of TBP in eukaryotic transcriptional mechanisms, and may pave the way for further investigations into the nature of these binding interactions. Further high-throughput assays and computational modeling are warranted to deepen our understanding and firmly establish the binding pattern specificity of gTBP. Ongoing studies on the binding between gTBP and ssDNA will provide us with valuable insights into both *Giardia* biology and the larger question of transcriptional control in eukaryotic systems.

## Experimental procedures

### *In silico* analysis of gTBP sequence and structure

We collected sequences for TBP orthologs from Uniprot for *G. intestinalis* (E2RU70), *S. cerevisiae* (P13393), *H. sapiens* (P20226), *E. histolytica* (P52653), *T. vaginalis* (A2DQT7), *P. falciparum* (Q8I440), and *L. tarentolae* (Q2TCV3). We then performed multiple sequence alignment using the Clustal Omega (version 1.2.4) program on the EMBL-EBI web server using default settings (56, 57).

Using AlphaFold 2.0, we generated a predicted structural model for gTBP based on its sequence (GiardiaDB: GL50803_001721) (58). Default settings were used for all parameters except for using the “monomer” model preset and the “full_dbs” database preset. The resulting model has an average per-residue confidence score or predicted local distance difference test (pLDDT) of greater than 90, with consistent scoring across all protein domains, indicating a high confidence for accurate structure prediction (58). We visualized both the predicted gTBP model and an experimentally determined model for *S. cerevisiae* TBP (PDB: 1YTB) using the PyMOL molecular graphics program. Phenylalanine residues identified as crucial for DNA binding, or their substitutions, are identified on the model images. To determine the approximate width of the DNA binding domain of gTBP, the distances between the alpha carbons corresponding to the conserved pair of phenylalanine residues in the stirrup of the TBP saddle structure were measured *in silico*. If the TBP is missing one or both phenylalanines in the TBP stirrup, then the alpha carbon of the substituted amino acids at these positions was used for this measurement.

To further characterize protein–DNA interactions in gTBP, we used AlphaFold3 to dock the protein to different nucleic acids (59). Five models are generated as the default option for each pair of protein and nucleic acid. The accuracy of the resulting models is quantified by two parameters, the interface predicted template modeling score (ipTM), which is associated with the accuracy of the relative positions of the protein and DNA in the complex, and the predicted template modeling score (pTM), which is associated with the overall accuracy of the structure of the complex. Both ipTM and pTM can vary between 0 and 1; high-quality models have ipTM scores above 0.8, while pTM scores above 0.5 indicate the model may resemble the actual structure. Examples of the TBP-DNA models obtained are shown in Fig. S3. A list of the TBPs and nucleic acids tested, along with their ipTM and pTM scores, is shown in File S5.

### Localization of *Giardia* TBP and other proteins by subcellular fractionation

Cultures of *G. intestinalis* WB C6 isolate (ATCC 50803) were grown to log phase (7.5 × 10^6^ cells/ml) in TYI-S-33 medium. The cells were collected and incubated in hypotonic buffer (10 mM HEPES, 1.5 mM MgCl_2_, 10 mM KCl, 0.2% IGEGAL C-630, pH 7.9) supplemented with 1× protease inhibitor cocktail and 10 μg/μl leupeptin for 30 min at 4 °C on a rotator. The sample was centrifuged at x2200 *g* for 20 min at 4 °C and the supernatant was collected as the cytosolic fraction. The pellet was resuspended in fresh hypotonic buffer and subjected to gentle grinding with a small plastic pestle within a microcentrifuge tube until all cells were lysed, as determined by examination of aliquots of the sample under a light microscope. The sample was centrifuged, and the pellet was washed three times with a hypotonic buffer. After the removal of the last wash, the pellet was resuspended in an alkaline RIPA buffer (75 mM NaOH, 80 mM glycine, 150 mM NaCl, 1.0% IGEPAL C-630, and 1.0% SDS, pH 10.6) supplemented with 1× protease inhibitor cocktail and 10 μg/μl leupeptin. This resuspension is designated as the organelle fraction.

Aliquots of the cytosolic and organelle fractions (∼40 μg protein/aliquot) were analyzed by immunoblotting with the following antibodies. A rabbit polyclonal antibody for gTBP was obtained by immunizing rabbits with GST-gTBP, followed by depletion of the antibodies against the GST portion of the protein on a GST column (commissioned from Bio Basic). Rabbit polyclonal antibodies against *Giardia* cytochrome *b*_5_ isotype III protein (GL50803_0033870) corresponding to amino acids 1 to 14 and against GiOR-1 (GL50803_0091252) corresponding to amino acids 1 to 165 were commissioned from GenScript (Piscataway). A rat polyclonal antibody against *Giardia* GAPDH (Gl50803_006687) was a gift from Soon-Jung Park of Yonsei University, Seoul, Korea. Rat polyclonal antibodies against the *Giardia* enolase (GL50803_0011118), and protein disulfide isomerase (GL50803_009413) were gifts from Pavel Doleźal of Charles University, Prague, Czech Republic.

### Immunofluorescence assay

The same antibodies used for the immunoblot analysis of the subcellular fractionation were used for immunofluorescence microscopy. For protein localization with the antibodies against cytochrome *b*_5_ isotype III (gCYTB5-III), GAPDH, and enolase, *Giardia* trophozoites were prepared by methanol fixation as previously described (60). The antibody dilutions are: gCYTB5-III at 1:2000, GADPH at 1:1000, and enolase at 1:1000.

For protein localization with antibodies against GiOR-1 (1:1000 dilution) and PDI2 (1:1000 dilution), *Giardia* trophozoites were prepared by paraformaldehyde fixation. Briefly, *Giardia* trophozoites from log phase cultures (7.5 × 10^6^ cells/ml) were allowed to adhere to poly-L-lysine coated coverslips. Paraformaldehyde was added to the coverslips to a final concentration of 2.4% and incubated at room temperature for 15 min. After quenching the formaldehyde by the addition of glycine to 10 mM, the solution was removed and replaced by a hybridization solution containing the antibody against the protein of interest and incubated at 4 °C overnight. The coverslips were then hybridized with FITC conjugated anti-rabbit antibody for the detection of the GiOR-1 antibody or Alexa488 conjugated anti-rat antibody for the detection of the PDI2 antibody. Coverslips were fixed to microscope slides with mounting medium containing DAPI (Vectashield, Vector Laboratories H-1200). Coverslips were sealed to the slides using nail polish. Slides were viewed using a Leica DM 6000B epifluorescence microscope and imaged using a Leica DFC 350 FX camera and LAS AF v.2.4.1 acquisition software.

The antibody against *Giardia* TBP was used at 1:500, 1:1000, and 1:5000 dilution in immunofluorescence assays with *Giardia* trophozoites prepared by methanol fixation and by paraformaldehyde fixation, but no specific immunostaining was observed.

### Epitope tagging of gTBP and its immunofluorescence localization in transfected *Giardia*

As our gTBP antibody did not work in immunofluorescence microscopy assays, we prepared a plasmid for ectopic expression from a native gTBP promoter of gTBP fused to two C-terminal StrepII tags in transfected *Giardia* trophozoites. Since the original *Giardia* expression plasmid (pAC) uses the strong constitutive ornithine carbamoyltransferase (OCT) promoter for expression of the tagged gene (61), we excised this promoter from the plasmid with *Hin*dIII and *Eco*RV and replaced it with the *Hin*dIII and *Eco*RV-digested PCR amplicon of the native gTBP promoter amplified from *Giardia* genomic DNA. Next, we digested this resultant plasmid (pAC-gTBP pro) with *Mlu*I and *Bam*HI and replaced it with the *Mlu*I and *Bam*HI digested PCR amplicon of the gTBP coding region amplified from *Giardia* genomic DNA. This resulted in the plasmid (pAC-gTBP pro+code).

*Giardia* was transfected by electroporation with the pAC-gTBP pro+code plasmid as previously described (62). The trophozoite cultures were then grown at 37 °C in TYI-S-33 medium with 10% serum and puromycin added to a final concentration to 50 μg/ml (63). The culture was grown and expanded until sufficient cells were available to prepare slides for immunofluorescence microscopy and to make frozen stocks of the culture for future use. *Giardia* trophozoites were prepared by methanol fixation as previously described (60) and then hybridized with an anti-StrepII antibody diluted at 1:1000 (GenScript A01732) followed by hybridization with a FITC-conjugated anti-rabbit antibody diluted at 1:200 (Jackson ImmunoResearch 115-095-00).

### Plasmid construction, protein expression, and purification of GST-gTBP

The coding region of *Giardia* TBP (GL50803_001721) was PCR-amplified from *Giardia* genomic DNA from the WB C6 isolate (ATCC 50803) with a forward primer incorporating a *Sal*I site and a reverse primer incorporating a *Xho*I site. The PCR product was purified and cloned in-frame behind the GST tag in pGEX-4T1 plasmid resulting in pGEX-4T1-gTBP.

Recombinant gTBP with an N-terminally attached GST-tag was expressed using *Escherichia coli* BL21 cells. In brief, *E. coli* cultures transformed with the pGEX-4T1-gTBP plasmid were grown in LB containing 100 μg/ml of ampicillin at 28 °C until the culture reached OD_600_ of ∼0.8 before it was induced by 1 mM isopropyl-beta-D-thiogalactopyranoside (IPTG). After induction, the culture was grown for an additional 16 to 24 h at 25 °C. Cells were collected, lysed, and loaded onto a GST-Trap column (Cytiva). The column was washed before the addition of glutathione elution buffer (20 mM reduced glutathione, 1 mM TCEP, 0.3 M NaCl) to collect the GST-tagged proteins into three 10-ml fractions. The eluate fractions were concentrated using a 10,000 molecular weight concentrator (Amicon) and the GST-tagged gTBP was exchanged into protein storage buffer (50 mM Tris-HCl pH 8.0, 5% (v/v) glycerol, 0.3 M NaCl, 0.3 mM MgCl_2_). Aliquots of the cell lysate, initial GST-Trap flow through, washes, and eluate were analyzed on a 14% SDS-PAGE gel to examine the relative purity of the gTBP prep (Fig. S4).

### Native PAGE analysis of GST-gTBP

To determine the oligomerization states of the GST-gTBP, the recombinant protein as well as GST alone was analyzed on native PAGE at pH 8.3. Samples of GST-gTBP (5 μg) and GST (5 μg) were prepared without the presence of SDS and loaded without prior heating onto a 4 to 20% gradient polyacrylamide gel (BioRad) and electrophoresed in running buffer lacking SDS. The gel was stained with Coomassie Blue after electrophoresis. Samples of BSA and rabbit IgG were also electrophoresed in adjacent lanes to give bands to use as approximate molecular weight markers. BSA has a monomeric form that is 66 kDa and a dimeric form that is 132 kDa, and rabbit IgG has a tetrameric form that is 150 kDa.

### Protein binding microarray, motif generation, and motif search in *Giardia* genome

For a high-throughput analysis of gTBP-DNA binding, protein binding microarrays (PBM) and subsequent data analysis were performed following the procedure described previously (26, 64). We used purified GST-gTBP protein at two different protein concentrations (1.5 and 3.0 μM), two different NaCl concentrations (0 and 0.15 M) in the hybridization reaction, and on two different types of single stranded PBM arrays (HK and ME) based on the De Bruijn sequence principle with unique compositions of DNA sequences. A total of eight PBM reactions with all combinations of these conditions were performed (see PBM details in File S3). Since sequences with an E-score ≥ 0.45 and a Z-score ≥ 6 are considered to reflect specific binding (65, 66), sequences with passing scores for both were aligned with the Clustal Omega multiple sequence alignment tool (67). The aligned sequences were then used with two online motif generation web servers, WebLogo and MEME (68, 69) to obtain position weight matrices for the preferential ssDNA-binding sequence of gTBP.

The sequences generated by WebLogo ([AC]NNGGGGNN[AC]), MEME ([ACG]N[ACT]GGGGN[ACG][ACG]), and GGGG were searched within the first 75 bp upstream of the ATG translation start codon for all genes excluding pseudogenes and three rRNA genes with corrupted data in the *G. intestinalis* WB isolate 2019 reference genome in GiardiaDB (https://giardiadb.org). The transcription initiation site has been determined experimentally for only a small number of *Giardia* genes and most of these sites are located 1 to 10 nucleotides upstream of the ATG start codon (21, 22, 27, 28). Therefore, the promoter region is expected to be within 75 bp upstream of the translation start codon for most *Giardia* genes. These motifs were searched in both strands of the upstream region where the (+) strand corresponds to the coding strand or the a-strand as defined in this study, and the (−) strand corresponds to the template strand or the b-strand as defined in this study. Results of these motif searches are in File S4.

To determine the distribution of different lengths of low-base stacking regions (LBSRs) in *Giardia* gene promoters, we downloaded 75 bp upstream sequences from the ATG start codon for all genes excluding pseudogenes and three rRNA with corrupted data (see details in Summary tab in File S6) in the *G. intestinalis* WB isolate 2019 reference genome in GiardiaDB (https://giardiadb.org). Using a custom Python script, we scanned both the coding (+) and noncoding (−) strands of each promoter region to identify the most likely TBP binding region, based on our previously described single-stranded base stacking energy model. Briefly, we searched for the longest contiguous LBSR characterized by low-energy nucleotides (C, T, and isolated A), and allowing 0 to 3 high energy points (HEPs) where each HEP is created by an isolated G (that is part of a GN or NG dinucleotide) or an AA dinucleotide. See Table 1 for base stacking energy values for each possible dinucleotide. Moreover, each HEP must be isolated so that it is surrounded by either a C, T, or isolated A. For instance, CT**G**A**G**TC would be scored as containing two HEPs, but CT**GG**TC and CT**G**AATC would not be allowed in a LBSR. If a HEP is found at the extreme 5′ or 3′ end of a LBSR, the LBSR length would be shorten in these cases to exclude the terminal HEP. When multiple candidate sequences of equivalent length were found in a single promoter, we selected the one with the lowest mean base stacking energy. The Python code for this analysis can be found in “genome_search.py” (https://doi.org/10.5281/zenodo.13826827). Subsequently, we generated split violin plots with Python Seaborn to show the distribution of the lengths of all predicted gTBP-binding regions, separated by DNA strand and maximum number of allowable HEPs.

### RNAcompete

We performed RNA pool generation, RNAcompete pulldown assays, and microarray hybridizations as previously described (70, 71, 72). Briefly, 20 pmoles of GST-tagged gTBP and RNA pool (1.5 nmoles) were incubated in RNAcompete assays in 1 ml of Binding Buffer (20 mM HEPES pH 7.8, 80 mM KCl, 20 mM NaCl, 10% glycerol, 2 mM DTT, 0.1 μg/μl BSA) containing 20 μl glutathione Sepharose 4B (GE Healthcare) beads (prewashed 3 times in Binding Buffer) for 30 min at 4 °C, and subsequently washed four times for 2 min with Binding Buffer at 4 °C (72).

### Electrophoretic mobility shift assays

We performed electrophoretic mobility shift assays (EMSAs) using purified GST-tagged gTBP or GST alone as a negative control (73, 74). Purified GST was a gift from Dr Logan Donaldson at York University. Oligonucleotides corresponding to the 50 to 60 bp sequence upstream of the gene of interest were used. In the case of the genes encoding glutamate dehydrogenase (GDH), histone H4, alpha-tubulin, and Ran, these upstream regions have been functionally identified as the promoter regions in previous luciferase reporter studies (9, 21, 22, 54) For other genes, these upstream regions are predicted to contain the promoter since previously characterized *Giardia* promoters are usually contained with 50 bp upstream of the start of the coding region. In this study, we designated the “a-strand” of the promoter region as the same strand as the coding or sense strand, while the “b-strand” is the non-coding or template strand. A list of all DNA sequences used in EMSAs is found in File S2.

Fluorescently labeled (5’Cy5) DNA probes and unlabeled DNA competitors were synthesized (Integrated DNA Technology). DNA probes and competitors were suspended in 10 mM Tris-HCl, pH 8.0, and their concentrations were determined by A_260_ readings on a LVis plate in a BMG Labtech FLUOstar Omega plate reader. Single-stranded DNA probes and competitors were individually heated at 90 °C for 10 min, and then the samples were immediately transferred to ice to minimize potential secondary structure formation before addition to binding reactions. For the double-strand DNA titration (Fig. 3), we annealed complementary strands—where only one of the strands is Cy5-labeled—in different molar ratios. We heated the complementary oligonucleotide pairs at 90 °C for 15 min in annealing buffer (50 mM Tris-HCl pH 8.0, 10 mM MgCl_2_, 50 mM NaCl) and then slowly cooled them at a rate of 5 °C/min until room temperature to promote duplex formation. An aliquot of binding buffer containing gTBP was added to each sample at the completion of this annealing procedure and then incubated on ice for 30 min before gel analysis.

Standard EMSA reactions contain 0.25 μg/μl of purified gTBP and 0.1 pmol/μl of Cy5-labelled probe DNA. In competitive EMSAs, unlabeled DNA were added to 20 pmol/μl, which is a 200-fold molar excess compared to the labeled DNA used as the probe. We incubated all reactions on ice for 30 min in binding buffer (10 mM Tris-HCl pH 8.0, 0.5 mM TCEP, 15 ng/μl sonicated salmon sperm DNA, 5% (v/v) glycerol, 1 μg/μl BSA, 0.15 M NaCl). We loaded the reactions without the addition of a tracking dye into non-denaturing agarose gels, as we noticed that common tracking dyes such as bromophenol blue and Orange-G interfere with the binding of gTBP to DNA. We used a ChemiDoc MP gel imager (BioRad) with DyLight 650 attachment for the direct detection of the fluorescent Cy5 dye conjugated to the DNA probe. Exposure times varied depending on the DNA probe used and are specified for each EMSA gel shown. The exposure time is chosen such that it is the maximum amount of time where no pixels in the bound protein-DNA bands are saturated, to permit accurate quantification.

For most EMSA reactions (Figs. 3, 4, 6, 8 and 9) we loaded the samples into a 2.5% agarose gel in 0.5× TB buffer (65 mM Tris pH 7.6, 22.5 mM boric acid) and electrophorized at 250 V for 25 min in a 4 °C cold room, adapted from (75). The EMSA reactions in Figures 7 and S10 were loaded into a 1% agarose gel in in 0.5× TBE buffer (65 mM Tris pH 7.6, 22.5 mM boric acid, and 1.25 mM EDTA) and electrophoresed at 130 V for 60 min at room temperature.

When specified, we determined the relative binding affinity of competitor DNA by quantifying the volumes of shifted protein-DNA bands using the densitometry feature in the Image Lab software suite (BioRad). To obtain relative binding affinity, we normalized the integrated volume for the band in the competitor reaction using the integrated volume for the band in the reaction containing only the DNA probe and purified gTBP without any competitors as the reference sample. The result of this normalization is a relative linear scale ranging from 0 (no competition/low binding affinity) to 1 (complete competition/high relative binding affinity).

### EMSAs with ssDNA probes and competitors prepared to promote and stabilize DNA secondary structures

Single-stranded DNA probes and competitors were individually prepared in annealing buffer (50 mM Tris-HCl pH 8.0, 10 mM MgCl_2_) containing 50 mM of either NaCl, KCl, LiCl, or without additional salt. Next, the DNA samples were heated at 90 °C for 15 min and slowly cooled at a rate of 5 °C/min until 25 °C to promote secondary structure formation. To prepare the control unannealed sample, the DNA was heated and immediately placed on ice. ESMA reactions were set up in our standard binding buffer (10 mM Tris-HCl pH 8.0, 0.5 mM TCEP, 15 ng/μl sonicated salmon sperm DNA, 5% (v/v) glycerol, 1 μg/μl BSA, 0.15 M NaCl) along with an aliquot of the annealed or unannealed DNA, an aliquot of gTBP, before incubation on ice for 30 min. For the competition with the telomere sequences in Figure 8*B*, individual DNA samples were placed in an annealing buffer (50 mM Tris-HCl pH 8.0, 10 mM MgCl_2_, 50 mM NaCl, 30 mM KCl), heated at 90 °C for 15 min and slowly cooled at a rate of 5 °C/min until 25 °C to promote secondary structure formation. ESMA reactions were set up in our standard binding buffer with an aliquot of the annealed or unannealed DNA, an aliquot of gTBP, and then incubated on ice for 30 min.

### Equilibrium dissociation constant determination

Estimated binding affinity was determined for all DNA probes used in this study *via* protein titration EMSAs to obtain quantitative values for equilibrium dissociation constants (*K*_D_) (76). A constant 0.1 pmol/μl of the specified DNA probe was incubated in individual reactions, as described above, with increasing amounts of purified gTBP from 0 to 2500 ng/μl. Imaging and densitometry were performed as described above, with the additional requirement that there were also no overexposed pixels in the bands of the free probes to permit appropriate quantification. *K*_D_ values were determined for the total amount of binding between gTBP and each of the four DNA probes used. Average values for the relative volume of the free probe at all concentrations (*n* = 4) were fit to the Hill equation using the nls (nonlinear least squares) function in R version 4.3.1.

### Single-stranded dinucleotide base stacking energy calculations

As a simple measure of overall structure and flexibility of single stranded DNA, we used the base stacking energy (*U*_S_) of each dinucleotide pair in the DNA sequence. We computed *U*_S_ values using the model developed by Chakraborty *et al.* (51) (Table 1). Using Eq. (1), we computed predicted *U*_S_ values for each possible dinucleotide in the 5′ to 3′ direction at 4 °C (277 K) (Table 1); where *h* and *s* are adjustable parameters relating to the enthalpy and entropy of stacking interactions (Table 1), *T* is the reaction temperature, *T*_*m*_ is the melting temperature of the dinucleotide (Table 1), and *k*_*B*_ is the Boltzmann constant.

(1) $$U_{S}=-h+k_{B}\left(T-T_{m}\right)s$$

To generate a per-nucleotide base stacking profile for an ssDNA sequence, we first used the values in Table 1 to determine the energy for each dinucleotide. Then, for each nucleotide, we took the average value of all overlapping dinucleotide base stacking energies. For instance, with the example sequence 5′ GACT 3′ the energy of the “G” nucleotide would simply be equal to the *U*_S_ of the dinucleotide GA (−4.95). The energy of the “A” nucleotide would be the average of the *U*_S_ of the dinucleotides GA (−4.95) and AC (−4.24), *i.e.* −4.60.

### *In silico* structural competitor generation

We generated *de novo* competitor ssDNA sequences using a custom Python script, accessible in “de_novo_generation.py” (https://doi.org/10.5281/zenodo.13826827). The script generates new sequences based on a given parent sequence. Generated sequences are the same length as the parent sequence, have a similar base stacking energy profile, yet are as dissimilar as possible in terms of their nucleotide sequences. Sequence similarity is determined between the original and generated sequences using the Needleman-Wunsch algorithm for the global alignment of similar-length sequences (77). Per nucleotide base stacking energy profiles are determined as described earlier, then similarity is determined using the root mean square error across all nucleotides, normalized by the range of possible base stacking energy values. (Equation 2) shows the “cost” function between two sequences as the product of the sequence alignment score and the normalized root mean square error of base stacking energies, where a lower score indicates similarity in base stacking energy profiles and dissimilarity in nucleotide sequences.

(2) $$\text{cost}=\left(\frac{\text{NW}\;\text{alignment}\;\text{score}}{\max\left(\text{NW}\;\text{alignment}\;\text{score}\right)}\right).\left(\sqrt{\frac{\sum_{i=1}^{\#\;\text{nts}}{\left({\text{BSE}\_\text{seq}1}_{i}-{\text{BSE}\_\text{seq}2}_{i}\right)}^{2}}{\#\;\text{nts}}}\cdot \frac{1}{\max\left(\text{BSE}\right)-\min\left(\text{BSE}\right)}\right)$$

Given an input sequence, the algorithm in the Python script derives new sequences that minimize the ‘cost’ function defined in (Equation 2). between the input and generated sequences. This is done by searching for the local sequence space around the input sequence through a randomized greedy process (stochastic local search) (78). In each iteration, a set of *n* new sequences are generated from the input sequence (or current best sequence) such that each nucleotide has a *p* probability of randomly mutating. The “cost” is calculated for all *n* sequences, and the sequence with the lowest cost is selected as the new “best sequence” (or the current best sequence is selected if none of the *n* sequences have a lower cost). This best sequence is then used as the input for the next iteration to generate new sequences. This iterative process is repeated, yielding a final sequence which maximizes the base stacking energy similarity and minimizes sequence similarity.

### Polynomial regression model used to predict relative gTBP binding scores for a given ssDNA sequence

Given a set of 36 sequences (File S2) and their relative binding scores, obtained as described above, we generated a model that aimed to predict the relative binding score based on each input sequence. The model we developed was a custom polynomial regression model using Nesterov gradient descent to optimize the parameters of the model (82). The code for the model can be found in “polynomial_regression.py” (https://doi.org/10.5281/zenodo.13826827). The parameters used for the regression analysis are listed in Table S1 and a complete description of the model can be found in Fig. S7.

## Data availability

The custom python code for *de novo* competitor ssDNA sequences and the polynomial regression model for the gTBP in the B-mode can be found in doi: 10.5281/zenodo.13826827. https://zenodo.org/records/13826827.

## Supporting information

This article contains supporting information (13, 14, 69, 70, 71, 79, 80, 81, 82, 83, 84, 85, 86, 87).

## Conflict of interest

The authors declare that they have no conflicts of interest with the contents of this article.

## Supporting information

File S1c

File S2cii
