# Supplementary material for: A TATA-box-binding protein binds single-stranded DNA in two modes: To poly(G) tracts and to flexible DNA regions
Source: J Biol Chem. 2025 Apr 27;301(6):108552. doi: 10.1016/j.jbc.2025.108552 (PMC12209930; doi:10.1016/j.jbc.2025.108552)
Supplement: File S1c [file mmc1.docx]

**Supporting Information for:**

**A TATA-box binding protein binds single stranded DNA in two modes: to poly(G) tracts and to flexible DNA regions**

Kieran Freitag^1^, Melanie Marlow^1^, Joella Joseph^1^, Robert Ta^1^, Jessica Krekhno^1^, Evan Schuett^1^, Ally Yang^2^, Debashish Ray^2^, Timothy Hughes^2^, Steven Rafferty^1^, and Janet Yee^1^*

1. Biochemistry and Molecular Biology Program, Trent University, Peterborough, ON

2. Department of Molecular Genetics, University of Toronto, Toronto, ON

*Corresponding author

**This File S1 contains:**

Figure S1: AlphaFold model confidence indicators for gTBP

Figure S2: Comparison of DNA binding pocket of TBPs from different species

Figure S3: Modelling of DNA binding to TBPs using AlphaFold 3

Figure S4: SDS-PAGE of GST-gTBP purification

Figure S5: RNACompete results

Figure S6: Oligomeric analysis of GST-gTBP on a native PAGE gel.

Figure S7: Overview of the training process for the polynomial regression model used to predict relative gTBP binding scores for a given ssDNA sequence.

Figure S8: EMSA of test sequences from polynomial regression analysis

Figure S9: Summary plots and graphs from polynomial regression analysis

Figure S10: EMSA with H2B-a and H4-a probes with homopolymer competitors and b-strand competitors.

**Table S1: Parameters used for regression analysis to predict gTBP binding**

Computed parameter weights and bias for the polynomial regression model to predict relative gTBP binding scores for ssDNA.

**Figure S1**

Alphafold model confidence indicators for gTBP. Left: ribbon structure of the model coloured according to per residue confidence (predicted local distance difference test, or pLDDT), where blue indicates a high confidence. Right: the predicted aligned error (PAE) for the model structure. Northwest quadrant (NW), aligned and scored residues within the amino-terminal half of the saddle; southeast quadrant (SE), aligned and scored residues within the carboxy-terminal half of the saddle; Northeast quadrant (NE), aligned residues within the amino terminal half of the saddle and scored residues within the carboxy-terminal half of the saddle; Southwest quadrant (SW) aligned residues within the carboxy- terminal half of the saddle and scored residues within the amino-terminal half of the saddle. Source: https://alphafold.com/entry/E2RU70.

**Figure S2**

Comparison of the saddle structures of TATA-binding proteins from different species, showing the distances between the pairs of alpha carbons corresponding to the highly conserved phenylalanines in the saddle (*e.g.* *S. cerevisiae* residues 116 and 207) and the stirrups (*e.g.* *S. cerevisiae* residues 99 and 190). The structure of *S. cerevisiae* TBP obtained by x-ray diffraction (xrd) corresponds to 1tbp.pdb (*1*) which does not have DNA bound, while the structure obtained by NMR corresponds to 1tba.pdb (*2*) and is an ensemble of 25 structures with the range of distance shown above; the average distance between residues 99 and 190 is 25.7 ± 0.5 Å and between residues 116 and 207 is 35.0 ± 0.5 Å. *H. sapiens* structures were both determined by x-ray diffraction for the saddle occupied by protein (6mzd.pdb, *3*) or by double-stranded DNA (1tgh.pdb, *4*). Structures for the protist TBPs were generated by Alphafold2 (*5,6*) using the following UniProtKB accession numbers: P52653 (*E. histolytica*), A2DQT7 (*T. vaginalis*), P32086 (*P. falciparum*), Q2TCV3 (*L. tarentolae*), E2RU70 (*G. intestinalis*). All structures were aligned to *G. intestinalis* TBP using the molecular graphics program PyMOL.

References for structures:

1. Chasman, D.I., Flaherty, K.M., Sharp, P.A., Kornberg, R.D. (1993) Crystal structure of yeast TATA-binding protein and model for interaction with DNA. *Proc Natl Acad Sci USA* 90: 8174-8178.
2. Liu, D., Ishima, R., Tong, K.I., Bagby, S., Kokubo, T., Muhandiram, D.R., Kay, L.E., Nakatani, Y., Ikura, M. (1998) Solution structure of a TBP-TAF(II)230 complex: protein mimicry of the minor groove surface of the TATA box unwound by TBP. *Cell* 94: 573-583
3. Patel, A.B., Louder, R.K., Greber, B.J., Grunberg, S., Luo, J., Fang, J., Liu, Y., Ranish, J., Hahn, S., Nogales, E. (2018) Structure of human TFIID and mechanism of TBP loading onto promoter DNA. *Science* 362. DOI: 10.1126/science.aau8872.
4. Juo, Z.S., Chiu, T.K., Leiberman, P.M., Baikalov, I., Berk, A.J., Dickerson, R.E. (1996) How proteins recognize the TATA box. *J Mol Biol* 261: 239-254
5. Jumper, J. *et al*. (2021) Highly accurate protein structure prediction with AlphaFold. *Nature*, 596: 583–589.. DOI: 10.1038/s41586-021-03819-2
6. Varadi, M. *et al*. (2023) AlphaFold Protein Structure Database in 2024: Providing structure coverage for over 214 million protein sequences. *Nucleic Acids Research*. DOI: 10.1093/nar/gkad1011

**Figure S3**

Modelling of DNA binding to TBPs using Alphafold 3. **(A)** Two models of Giardia TBP bound to single-stranded DNA containing the CT-rich central sequence in the b-strand of the histone H4 promoter (see H4-C sequence in Figure 8). The two models chosen are the two most different in their conformations among five default AlphaFold3 models generated. **(B)** Model of human TBP bound to the double-stranded TATA sequence TATATAAT found in the Adenovirus E1B gene (see Adenovirus E1B TATA sequence in Fig. 4). **(C)** Model of human TBP bound to single-stranded TATA sequence TATATAAT. DNA coloured A=yellow, T=cyan, C=magenta.

**Figure S4**

Representative SDS-PAGE gel showing various steps in the GST-gTBP purification. FT is the flowthrough fraction. Lanes labelled F1, F2, and F3 represent the first, second, and third fractions of the final purified protein. Only the concentrated F1 sample was used in our study. The last lane contains 5 µg of the purified GST alone.

**Figure S5**

RBFOX1 gTBP


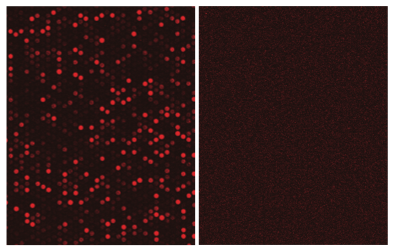


Interrogation of gTBP RNA-binding activity. To analyze gTBP RNA-binding activity, we used RNAcompete (Ray et al., 2009, 2013, 2017). During the RNAcompete assay, GST-tagged gTBP or RBFOX1 (control RBP) were incubated with a diverse, custom designed RNA library containing ~241K unique RNA probes. Bound RNA was eluted, fluorescently labeled with Cy5, hybridized to custom Agilent 244K microarrays, and subsequently scanned using an Axon microarray scanner. Representative microarray regions showing fluorescent signals (red “spots”) corresponding to Cy5-labeled RNA bound by either RBFOX1 or gTBP are shown in the above panels and illustrate the relative levels of RNA-binding (or lack of) by RBFOX1 and gTBP. Microarray images were magnified using Imagene 8.0.

**Figure S6**

Oligomeric analysis of GST-gTBP on a native PAGE gel. To determine the oligomerization states of the GST-gTBP, the recombinant protein as well as GST alone was analyzed on a native PAGE gel at pH 8.3. Samples of BSA and rabbit IgG were electrophoresed in adjacent lanes to give bands to use as approximate molecular weight markers. The expected oligomeric forms of each protein sample and their corresponding expected molecular weights are given in the table.

**Figure S7**

Overview of the training process for the polynomial regression model used to predict relative gTBP binding scores for a given ssDNA sequence.

The model generates predicted binding scores for a given nucleotide sequence, based on the best subsequence of a specified length. For instance, the range of lengths we used in our final model was 18 to 24 nucleotides. Therefore, to predict binding for a sequence of 60 nucleotides, the model is applied for every possible subsequence between 18 and 24 nucleotides along the entire 60 nucleotide stretch, and the results of the highest scoring subsequence are chosen to represent the sequence as a whole. This process reflects how the binding of a protein along a large sequence will be determined by the exact location in which it binds (i.e., the ‘binding’ subregion).

Predicted binding affinity of a subsequence is based on three parameters: (1) the length of the subsequence, (2) the average base stacking energy of the subsequence, and (3) the number of G nucleotides in the subsequence. The values of each of these three parameters are clamped to pre-determined maximum and minimum values and normalized from 0 to 1. The length is clamped between 18 and 24, the average base stacking energy from -4.6 to -4.2, and the number of G nucleotides from 0 to 3. We manually chose these three parameters and their ranges based on subjective observations of how they seem to affect binding affinity.

In essence, this training process uses a momentum-based (Nesterov) gradient descent algorithm to iteratively improve the polynomial weights for each input parameter to more closely predict relative binding scores. This training process is done in two stages: (1) a ‘search’ phase and (2) a ‘train’ stage. In the first ‘search’ phase, gradient descent is performed at many different randomized starting values for the model weights and bias (500 initial states was used). By repeating gradient descent from many randomized initial states, we increase the probability that the global minimum is found, i.e., the best possible weights and bias to predict binding scores. The parameters for gradient descent in this stage are set so that a rough search is done (iterations = 300, learning rate = 0.1, momentum = 0.9). In the second ‘train’ phase, gradient descent is performed again on the weights and bias that yielded the best average score in the initial ‘search’ phase. The parameters for gradient descent were selected for a fine determination of the local minima (iteration = 100000, learning rate = 0.025, momentum = 0.8).

We chose a polynomial model of degree 4 (i.e., there are four weights for each parameter, one for each polynomial degree) to accommodate potential nonlinear relationships between each parameter and the output binding score. This would capture the appropriate relationship if, for example, low average base stacking energy relates to high binding affinity, medium energy with low affinity, and high energy with high affinity. A linear regression model would fail to capture this relationship. We also chose Nesterov batch gradient descent for the optimization algorithm as it is efficient and accurate in determining local minima (Ruder, 2016).

To validate the model, we compared the predicted relative binding affinities to the actual EMSA-derived binding affinities for 12 novel sequences (40 nucleotides long) found from Giardia gene promotors (Figure S8 for EMSA). These sequences were initially chosen to represent a broad range of predicted affinity values in an effort to see if the model is valid across the possible range of outputs. However, the selection was done on a very early iteration of the model which does not correlate with the values produced by the final model presented here. As such, the novel test sequences can be seen as a pseudo-randomized sample of several upstream regions across various genes in the Giardia genome. Both coding and non-coding sequences are equally represented in this sample set. Exact genes, relative upstream locations, and strand information can be found in File S2.

**Figure S8**

gTBP differentially binds to a set of test sequences from *Giardia* promotor sites (coding and non-coding strands). (**A**) Sequences and base stacking energy profiles for the set of DNA competitor sequences. Blue regions indicate low base stacking (U_s_ less than approx. –4.8) and yellow regions indicate high base stacking (U_s_ greater than approx. –4.5). (**B**) Representative EMSA (1 of 2) with labelled H4-b DNA probes against unlabelled competitor sequences. (**C**) Similar to B, using H2B-b labelled probes. (**C**) Quantification of binding to competitors on EMSAs for both DNA probes (n=2 each; n=4 in total). Relative strength of competition is determined with respect to the lane containing only DNA probe and gTBP. Relative binding value ranges from 0 (no competition/low relative binding affinity) to 1 (complete competition/high relative binding affinity). Error bars represent standard deviation.

**Figure S9**

An incomplete polynomial regression model to determine gTBP binding to ssDNA based on different features of the sequence. (**A**) Polynomial graphs showing the output weights for given inputs values for the three different features selected. Dots along the polynomial line indicate that the input values for that feature are discrete, i.e., there are no valid intermediate input or output values. (**B**) Residual plots for the correlation between actual and predicted binding score for the set of training ssDNA sequences and for the set of test sequences. Error bars represent standard deviation across 4 EMSA samples (2x H4-b probe, 2x H2B-b probe). Training sequences were used to generate the polynomial weights for the three features used in the regression model. Test sequences were selected from *Giardia* promotor regions in order to validate the accuracy of the model. R-squared (R^2^) values are shown for each set of sequences.

Results from the polynomial regression model we developed to predict relative binding affinity from ssDNA sequence did not predict the binding ability of 12 test sequences. Limitations may be attributed to fundamental aspects of nonlinear regression systems, such as the difficulty in differentiation between global and local minima when training the model to best fit the input data (Motulsky & Ransnas, 1987; Ruder, 2016). However, it is more likely that the input parameters we manually chose were insufficient to fully predict binding affinity. We chose subregion length (LBSR), average stacking energy, and the number of G nucleotides as input parameters with equal weighing based on general observations on how these variables impact binding affinity via the sets of ssDNA sequences we tested. Ideally, to eliminate human bias in determining features, future work could make use of modern deep learning approaches via the use of convolutional neural networks, or other predictive networks; reviewed in (Alzubaidi et al., 2021). Such predictive tools could be trained on input data representing raw sequence and base stacking information, eliminating the need to manually select individual features. We were initially reluctant to using these techniques due to the “black box” phenomena, wherein the process for how these networks predict output variables from input data is convoluted and uninterpretable. Many recent efforts, however, have been made to develop processes to improve the interpretability of these models (Dong et al., 2017; Fernando et al., 2019; Wu et al., 2021), making such deep networks a viable and compelling next step.

**Figure S10**

Testing A-mode of gTBP binding. **(A)** EMSAs of the a-strand of the H4 and H2B probes with 20-mers of either polyA, polyC, polyT and polyC as competitors (360 sec exposure). **(B)** Sequences of DNA probes with the PBM motif highlighted in blue. **(C)** EMSAs of the a-strand of the H4 and H2B probes competed with cross complementary sequences and *de novo* generated sequences matching the base stacking energy profiles of the b-strands of H4 and H2B from Figure 9 (45 sec exposure). **(D)** Sequences of DNA probes and competitors with the PBM motif highlighted in blue.

**Table S1**

Computed parameter weights and bias for the polynomial regression model to predict relative gTBP binding scores for ssDNA sequences following the procedure outlines in the Methods.

| Parameter weights (*w*_i,j_) | *x*^0^ | *x*^1^ | *x*^2^ | *x*^3^ |
| --- | --- | --- | --- | --- |
| Avg. base stacking energy | -0.14082 | 1.52208 | 0.83199 | -1.13496 |
| Subregion length | 0.13739 | 0.42873 | -0.15083 | -1.35024 |
| Number of G nucleotides | -0.67488 | 3.21343 | -1.08492 | -0.86207 |
| Constant bias (*b*) | -0.16857 | | | |
